# Glycemic traits and colorectal cancer survival in a cohort of South Korean patients: A Mendelian randomization analysis

**DOI:** 10.1002/cam4.7084

**Published:** 2024-03-13

**Authors:** So Yon Jun, Sooyoung Cho, Min Jung Kim, Ji Won Park, Seung‐Bum Ryoo, Seung Yong Jeong, Kyu Joo Park, Aesun Shin

**Affiliations:** ^1^ Department of Preventative Medicine Seoul National University College of Medicine Seoul Republic of Korea; ^2^ Department of Surgery Seoul National University College of Medicine Seoul Republic of Korea; ^3^ Cancer Research Institute Seoul National University Seoul Republic of Korea; ^4^ Medical Research Center, Genomic Medicine Institute Seoul National University College of Medicine Seoul South Korea; ^5^ Integrated Major in Innovative Medical Science Seoul National University College of Medicine Seoul South Korea

**Keywords:** colorectal cancer, glycemic traits, Mendelian randomization analysis, Type 2 diabetes

## Abstract

**Background:**

Clinical diabetic traits have been reported to be associated with increased colorectal cancer (CRC) risk in observational studies. Using the Mendelian randomization (MR) analysis method, we examined the causal association between glycemic traits, such as fasting glucose (FG), fasting insulin (FI), and glycosylated hemoglobin A1c (HbA1c), and survival in a cohort of CRC patients.

**Methods:**

We conducted a two‐sample MR analysis among a cohort of patients with locally advanced CRC at Seoul National University Hospital. Single‐nucleotide polymorphisms robustly associated (*p* < 5 × 10^−8^) with the three glycemic traits were obtained from the Meta‐Analyses of Glucose and Insulin‐related traits Consortium, Asian Genetic Epidemiology Network, and Korea Biobank Array. Three‐year and 5‐year overall survival (OS) and progression‐free survival (PFS) were used as outcomes. Survival analysis was conducted using subgroup analysis by cancer stage and subsite in a multivariate Cox proportional hazards model adjusted for age and sex to examine whether glycemic traits affected survival.

**Results:**

A total of 509 patients were included in our final analysis. MR analysis showed that HbA1c levels were associated with poor 3‐year OS (*β* = 4.20, *p* = 0.02). Sensitivity analyses did not show evidence of any violations of the MR assumptions. In the cancer subgroup analysis of the Cox proportional hazards model, pooled hazard ratios for FG were significantly associated with poor 3‐year OS and PFS regardless of cancer stage. FI was not significantly associated with any 3‐year survival endpoints. Among Stage III patients, three glycemic traits were significantly associated with both 5‐year OS and PFS. Location‐specific subgroup analysis showed a significant association between three glycemic traits and 5‐year PFS in patients with left‐sided colon cancer. FG was associated with poor 3‐year survival for colon cancer but not rectal cancer.

**Conclusions:**

Our results suggest that FG and HbA1c could be used to predict prognosis in CRC patients. Lifestyle and/or pharmacological interventions targeting glycemic traits could help improve survival for CRC patients.

## 1 BACKGROUND

Colorectal cancer (CRC) is the third most commonly diagnosed cancer across the globe and is the second most common leading cause of cancer death.^1^ In South Korea, CRC is the third most common cancer for both women and men.^2^ A variety of modifiable risk factors, such as diet, obesity, hormonal, and developmental factors are thought to be associated with CRC risk and progression.^3^ Identifying risk factors involved in CRC prognosis may help improve cancer management in patients with locally advanced CRC.

The role of metabolic homeostasis in the pathogenesis of CRC has been of particular interest in recent years. Several studies have found diabetes to be associated with increased CRC risk across different ethnicities.^4^, ^5^, ^6^ Diabetic traits such as hyperglycemia and hyperinsulinemia have been reported to increase the risk of CRC in both diabetic and nondiabetic patients alike,^7^ suggesting that there exists an association between CRC and diabetic traits. In particular, CRC and Type 2 diabetes (T2DM) share pathophysiological pathways involving hyperglycemia, hyperinsulinemia, and insulin resistance through the regulation of insulin‐like growth factor (IGF) signaling pathways.^8^ Previous studies have also reported that hyperglycemia was independently associated with CRC mortality regardless of diabetic status in both European and South Korean patients.^7^, ^9^, ^10^

While many epidemiological studies suggest that there exists an association between CRC risk and glycemic traits, the clinical impact of glycemic traits on CRC prognosis has yet to be elucidated.^11^, ^12^, ^13^ Earlier literature has reported inconsistent results, which may arise from the presence of unmeasured confounders and potential biases that are inherent to observational studies.^14^ Mendelian randomization (MR) allows for a means to observe causal effects of an exposure on an outcome of interest without confounding or reverse causality.^15^ By utilizing single‐nucleotide polymorphisms (SNPs) as genetic instruments, the MR approach provides a model for analyzing the effects of glycemic traits on CRC survival outcomes.

In the present study, we conducted a two‐sample MR analysis to assess the potential causal association between glycemic traits and CRC survival outcomes in a cohort of South Korean patients. It is worthwhile to examine glycemic traits such as fasting glucose (FG), fasting insulin (FI), and glycosylated hemoglobin A1c (HbA1c) in a South Korean cohort, as most studies have been conducted using European populations. We also addressed whether the effect of glycemic traits on CRC survival differs by CRC stage and subsite; since the pathology, clinical presentations, and prognosis of left‐sided colon cancer (LCC), right‐sided colon cancer (RCC), and rectal cancer are different.^16^

## 2 METHODS

### 2.1 Study population and data collection

The study population was obtained from a prospective patient cohort from Seoul National University Hospital (SNUH) that consisted of 960 CRC cases. Patients who received an operation from May 2014 to June 2017 at SNUH were enrolled in the cohort. Clinical and demographic characteristics were obtained from the SNUH Electronic Medical Record and included cancer subtype (right‐sided colon, left‐sided colon, and rectum), stage, tumor size, and treatment type. Clinical data regarding glycemic levels and diabetic‐parameters, such as treatment and duration of diabetes, were not included in the cohort data. Survival status was also available up to 2020 by following up with patients during scheduled outpatient visits. Genotyping was performed using a customized SNP microarray chip, the Koreachip, that was developed by the Korea Biobank Array (KBA).

**FIGURE 1:**
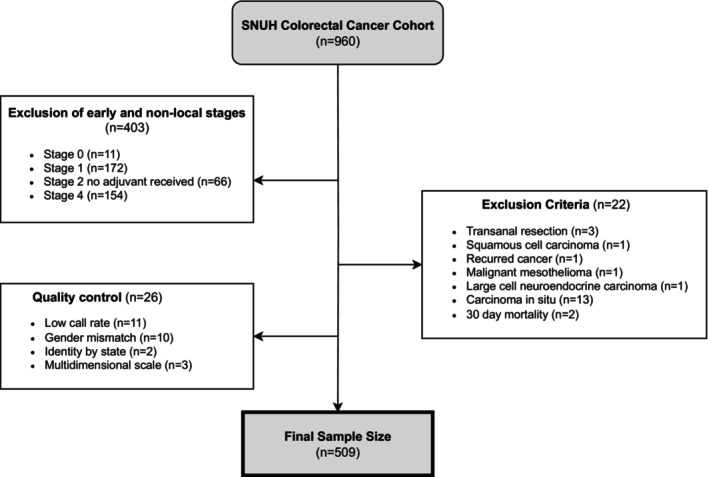
Flow chart describing the study population with exclusion criteria and quality control for the Seoul National University colorectal cancer cohort. SNUH, Seoul National University Hospital.

High‐risk stage II patients and stage III patients were included in our analysis of locally advanced CRC. According to the National Comprehensive Cancer Network Guideline, high‐risk stage II cancer patients are defined as those with poorly differentiated histology, bowel perforation, <12 examined lymph nodes, and lymphovascular invasion or perineural invasion. We excluded 22 patients who met the exclusion criteria (metachronous cancer, nonadenocarcinoma, and mortality within 30 days after surgery). Sample quality control excluded an additional 26 patients. A total of 509 patients were included in the final analysis (Figure 1). The outcome of interest was CRC prognosis, which was measured as 3‐year and 5‐year overall survival (OS) and progression‐free survival (PFS). OS was defined as the point from the date of operation until death. PFS was defined as the point from date of operation until first recurrence or death by any cause.

### 2.2 Genetic data and instrument selection

Genetic variants are used as instruments in MR analysis to examine the causal association between modifiable exposures and disease outcomes. Our exposures of interest were FG, FI, and HbA1c, while our outcome of interest was CRC survival. Directed acyclic graphs were drawn to show the potential pathways of causal association for our MR analysis (Figures S1 and S2).

Three key instrumental variable (IV) assumptions must be met to perform MR: instruments are associated with exposure; instruments are independent of confounders; instruments influence outcome only through the exposure. SNPs with statistically significant effect sizes validate the first MR assumption and the use of SNPs as instruments validates the second assumption, as genetic variants are randomly assorted at conception. Previously published GWAS datasets have already identified SNPs associated with glycemic traits. We utilized summary statistics from large‐scale GWAS datasets, as effect sizes may be overestimated if summary statistics from a smaller study population are used (the Beavis effect).^17^, ^18^ Specifically, we utilized summary statistics (e.g., chromosomal location, marginal effect sizes, standard errors, effect alleles, and other alleles) from the Meta‐Analyses of Glucose and Insulin‐related traits Consortium (MAGIC) to select SNPs with a genome‐wide statistical significance threshold (*p* < 5 × 10–8). Effect sizes in the MAGIC datasets were adjusted for sex and age but not adjusted for BMI. Variants for FG and FI were identified from the Metabochip replication datasets, which included 133,010 individuals of European ancestry without diabetes.^19^, ^20^ HbA1c‐associated variants were identified from an ancestry‐specific and transethnic METAL dataset that included 20,838 East Asian individuals.^21^

The selected SNPs from MAGIC were then compared to the Asian Genetic Epidemiology Network (AGEN) database^22^ to ensure that valid instruments among East Asian ancestry were selected. We also utilized recent findings from the KBA GWAS,^23^ which contained a total of 833,000 SNPs from 125,872 Korean individuals genotyped with a customized SNP microarray. Finally, the selected genetic instruments from both MAGIC and KBA were clumped (*R* ^2^ = 0.001 and window size = 10 MB) using an East Asian population reference to ensure that the selected SNPs were independent. Removal of SNPs with linkage disequilibrium (LD *R* ^2^ < 0.001) is an important step in improving the strength of MR analysis by ensuring that only independently, randomly assorted SNPs were selected as instruments^24^ (Tables S1 and S2 provides the full list of exposure‐associated SNPs used and their summary statistics).

### 2.3 Statistical analysis

#### 2.3.1 Mendelian randomization

A two‐sample MR analysis was conducted using the TwoSampleMR R package (version 0.5.5)^25^ for all 3‐year and 5‐year survival endpoints. Estimates of the association between individual genotyped SNPs and each survival outcome were calculated by performing a logistic regression analysis using effect sizes (*β*) from MAGIC and KBA summary statistics. Wald ratios were then calculated to estimate the causal effect size of each individual SNP. The fixed‐effects inverse‐variance weighted (IVW) method was used to estimate the causal effect of each set of glycemic trait‐associated SNPs. The IVW method uses a meta‐analysis approach to combine Wald ratios and is a reliable means of using summary MR data as long as variants are mutually independent.^26^, ^27^ We also performed additional MR–Egger, weighted median, simple mode, and weighted mode methods to test the robustness of the IVW estimates. Odds ratios (ORs) were also calculated to examine whether a 1‐SD increase in log genetically predicted glycemic trait levels was associated with OS and PFS.

Sensitivity analyses were performed to test the strength of our MR analyses and account for potential sources of biases such as pleiotropy and reverse causation. The IVW method assumes that the IV assumption is upheld and therefore that none of the IVs used exhibit horizontal pleiotropy. To test for potential unbalanced pleiotropic associations, we used the MR–Egger regression method as a sensitivity analysis. The MR–Egger method not only shows violations of the IV assumption but also provides an estimate of the causal effect under a weaker INstrument Strength Independent of Direct Effect (InSIDE) assumption. A nonzero MR–Egger intercept suggests that the IVW estimate is biased and that there is a significant pleiotropic effect between genetic variants.^28^ Heterogeneity was also tested with Cochran's *Q* test using MR–Egger and IVW estimates to determine which method was more accurate in estimating the true causal effect.^26^ Leave‐one‐out (LOO) analysis was conducted to assess whether an association was disproportionately influenced by an individual SNP. Our results were summarized into scatter plots, forest plots, and funnel plots. We estimated study power a priori using the mRnd online power calculation tool assuming an *α* of 0.05 for each factor and had sufficient power (>80%) to detect the effect size of each glycemic trait.^29^

#### 2.3.2 Survival analysis

We performed subgroup analysis by cancer stage (Stages II and III) and subsite (RCC, LCC, and rectal) using a Cox proportional hazard model adjusted for sex and age as a continuous variable to estimate adjusted hazard ratios (HRs) and 95% confidence intervals (CIs). We also pooled HRs for each SNP according to which of the three glycemic traits they were associated with to determine the prognostic impact of glycemic traits on survival endpoints. Schoenfeld residual plots were used to assess the proportional hazard assumption of our Cox model. *p* Values were two sided, and *p* < 0.05 was considered statistically significant. All statistical analyses were conducted using R (version 4.0.3).

## 3 RESULTS

### 3.1 Characteristics of the SNUH CRC cohort

Table 1 details the baseline characteristics of the SNUH CRC cohort. A total of 509 CRC patients were identified during a median follow‐up of 4.64 years, among which 186 were Stage II and 323 were Stage III cases. There were 101 patients who were diagnosed with diabetes, and 215 were diagnosed with hypertension at baseline. The median age was 62 years, and the median BMI was 23.6. For 3‐year survival, we observed 51 OS events and 134 PFS events. For 5‐year survival, 362 OS survival events and 375 PFS events were observed. Differences in baseline characteristics depending on diabetic status were detailed (Table S1). A higher proportion of diabetic patients were male compared to nondiabetic patients (74% vs. 52.7%; *p* < 0.001) and hypertension was significantly higher in diabetic patients (*p* < 0.001).

**TABLE 1:** Baseline characteristics of the SNUH colorectal cancer cohort.

Characteristics	*n* = 509
Sex, *n* (%)	
Male	289 (56.8)
Female	220 (43.2)
Age, median (IQR)	62 (46, 78)
BMI, median (IQR)	23.6 (19.3, 28.0)
Diabetes, *n* (%)	
No	408 (80.2)
Mild	100 (19.6)
Severe (end organ damage)	1 (0.2)
Hypertension, *n* (%)	215 (42.2)
Histologic grade, *n* (%)	
1	462 (90.8)
2	39 (7.7)
N/A	8 (1.6)
Stage, *n* (%)	
2	186 (37)
3	323 (63)
Enrollment year, *n*	
2014	170
2015	223
2016	77
2017	38
Median follow‐up, years	4.64

Abbreviations: IQR, interquartile range; *n*, number; SNUH, Seoul National University Hospital.

### 3.2 Causal association between glycemic traits and survival

**FIGURE 2:**
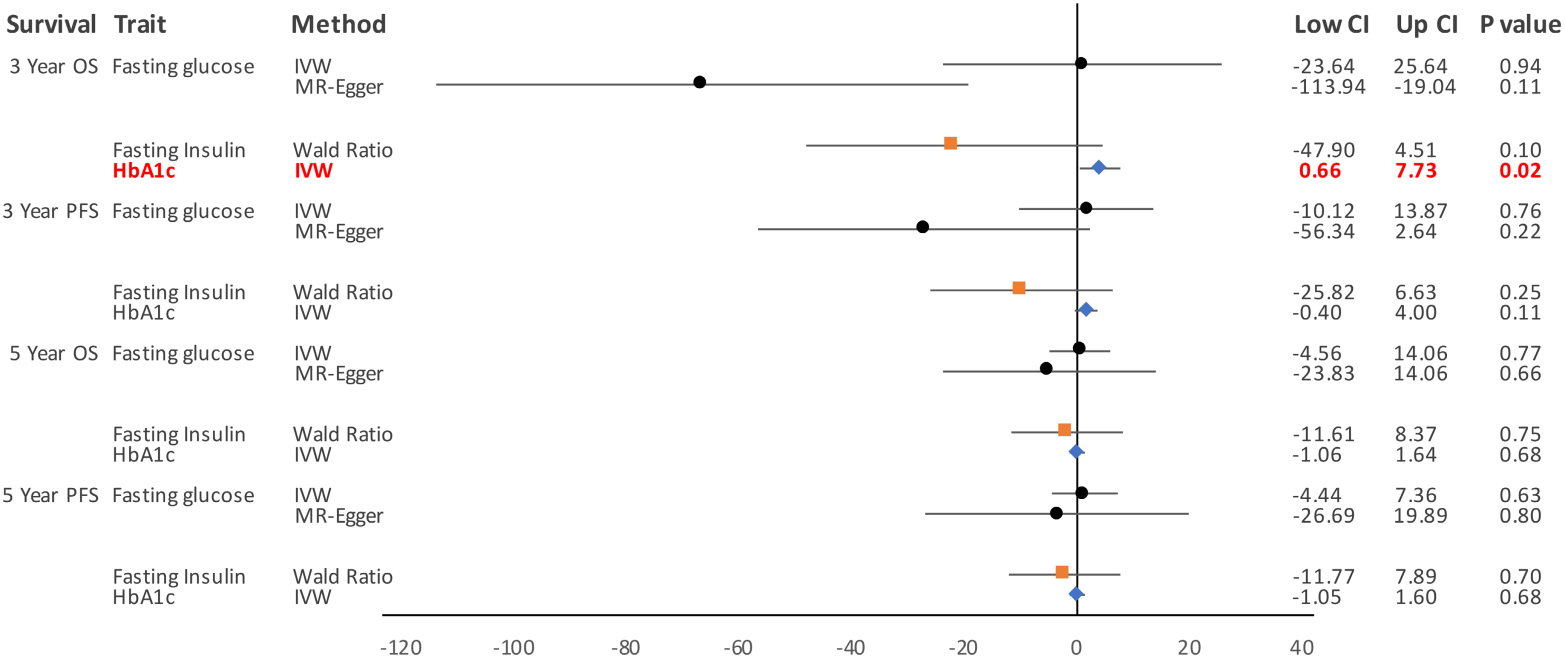
Forest plot shows causal effect size values and their 95% confidence interval by Mendelian randomization method used for each glycemic trait. CI, confidence interval; IVW, inverse‐variance weighted; OS, overall survival; PFS, progression‐free survival.

A total of seven SNPs was selected as instruments for our MR analysis; four were associated with FG, one with FI, and two with HbA1c (Table 2). No significant associations were found between Wald ratios calculated for each individual SNPs and all survival endpoints (Table S2). Additional MR analysis methods were performed for FG‐associated SNPs as there were more than two SNPs available. Figure 2 shows the causal effect sizes of each glycemic trait on each survival endpoint according to the MR method used. We did not find any significant associations between FG and any survival endpoints but did observe that the direction of the causal effect was different for IVW and MR–Egger estimates in regards to 3‐year OS (Figure 2). While the MR–Egger estimate (*β* = −66.48, se = 24.21, *p* = 0.11) for 3‐year OS was not statistically significant, sensitivity analyses showed that the MR–Egger estimate was more appropriate than the IVW estimate (Figure 2; Table S5).

**TABLE 2:** SNPs and their characteristics utilized for Mendelian randomization analysis.

SNP rsID	Chr	EA	OA	*β*	SE	*p*	Database
Fasting glucose (*n* = 4)							
rs10814916	9	C	A	0.015819	0.002156	2.26E‐13	MAGIC Metabochip
rs11558471	8	A	G	0.028879	0.002277	7.80E‐37	
rs7903146	10	T	C	0.021965	0.002378	2.71E‐20	
rs9368222	6	A	C	0.014251	0.002331	1.00E‐09	
Fasting insulin (*n* = 1)							
rs17036328	3	C	T	−0.021	0.003	3.59E‐12	MAGIC Metabochip
HbA1c (*n* = 2)							
rs1799884	7	T	C	0.1166	0.0081	1.66E‐47	Korea Biobank Array
rs2233580	7	T	C	0.1031	0.0124	9.27E‐17	

Abbreviations: Chr, chromosome; EA, effect allele; Hba1c, hemoglobin A1c; MAGIC, Meta‐Analyses of Glucose and Insulin‐related traits Consortium; *n*, number; OA, other allele; SNPs, single‐nucleotide polymorphisms.

The causal estimates we obtained for FI were not statistically significant but showed a consistent negative association with all survival endpoints (Figure 2). Additional MR methods in addition to the Wald ratio as well as sensitivity analyses were not performed as only one FI‐associated SNP was available for analysis. HbA1c was significantly associated with poor 3‐year OS by using the IVW method (*β* = 4.20, se = 1.80, *p* = 0.02) (Figure 2; Table S2). Although not significant, the two HbA1c SNPs (rs1799884, rs2233580) each showed positive causal associations with 3‐year OS which was consistent with the IVW estimate.

### 3.3 Colorectal cancer survival by cancer stage

Glycemic traits affected survival endpoints differently by cancer staging (Table 3). We found significant associations between FG and 3‐year survival endpoints. FG was associated with both poor Stage III 3‐year OS and PFS (HR >1). HbA1c was also associated with poor 3‐year OS (HR = 2.24, 95% CI = 1.79–2.70, *p*<0.0001) and PFS (HR = 1.47, 95% CI = 1.18–1.76, *p* < 0.0001). FI was only associated with poor Stage III OS (HR = 2.26, 95% CI = 1.18–4.31, *p* = 0.01). We did not find any significant associations between the three glycemic traits and any 5‐year survival endpoints for any stage.

**TABLE 3:** Association between glycemic traits and survival endpoints by cancer stage using the multivariate Cox proportional hazards model adjusted for age and sex.

	3‐year survival			5‐year survival		
	All (95% CI)	Stage II (95% CI)	Stage III (95% CI)	All (95% CI)	Stage II (95% CI)	Stage III (95% CI)
No. of cases	509	186	323	509	186	323
*Overall survival*						
No. of events	51	12	39	362	129	233
Fasting glucose	**1.57 (1.28,1.87)**	0.43 (‐0.33, 1.19)	**2.33 (1.98, 2.67)**	1.07 (0.97, 1.18)	0.95 (0.76, 1.14)	1.13 (0.99, 1.27)
Fasting insulin^a^	1.58 (0.91, 2.73)	0.43 (0.11, 1.59)	**2.26 (1.18, 4.31)**	1.03 (0.84, 1.28)	0.97 (0.68, 1.38)	1.07 (0.82, 1.39)
HbA1c	**1.59 (1.20, 1.98)**	0.44 (‐0.48, 1.37)	**2.24 (1.79, 2.70)**	1.03 (0.88, 1.18)	0.97 (0.72, 1.22)	1.06 (0.88, 1.24)
*Progression‐free survival*						
No. of events	134	93	41	375	131	244
Fasting glucose	**1.25 (1.06, 1.43)**	0.82 (0.48, 1.16)	**1.51 (1.29, 1.73)**	1.08 (0.97, 1.19)	0.94 (0.76, 1.13)	0.94 (0.81, 1.08)
Fasting insulin^a^	1.22 (0.87, 1.72)	0.81 (0.43, 1.52)	1.46 (0.97, 2.20)	1.04 (0.85, 1.28)	0.95 (0.67, 1.36)	1.09 (0.85, 1.41)
HbA1c	1.22 (0.98, 1.46)	0.78 (0.33, 1.24)	**1.47 (1.18, 1.76)**	1.03 (0.89, 1.18)	0.94 (0.69, 1.19)	1.08 (0.90, 1.26)

Abbreviations: CI, confidence interval; HR, hazard ratio; Hba1c, hemoglobin A1c; SNP, single‐nucleotide polymorphism.

Bolded values were statistically significant (*p* < 0.05) as the values in the parantheses represent the 95% confidence interval.

^a^ Pooled fasting insulin has the same values as the SNP rs17036328 as it was the only SNP available for analysis.

We did not find any associations between individual SNPs and 3‐year OS or PFS in Stage II CRC (Table S7). All SNPs (rs17036328, rs9368222, rs1799884, rs2233580, rs11558471, and rs10814916) excluding one (rs7903146), were associated with poor 3‐year OS in Stage III patients. No significant associations were found between all SNPs and 5‐year OS (Table S8). FG‐associated rs7903146 was associated with poor 5‐year PFS (HR = 1.52, 95% CI = 1.05–2.19, *p* = 0.03). Sensitivity analyses were performed for our stage‐subgroup analysis Cox model using the Schoenfeld residual test. Schoenfeld residual plots showed no significant correlation between outcomes and time, validating the proportional hazards assumption (Figure S5).

### 3.4 Colorectal cancer survival by cancer location

Survival was affected by glycemic traits differently according to cancer site as well (Table 4). FG was significantly associated with poor 3‐year OS and PFS for all sites excluding the rectum. Three‐year survival was worse in RCC (OS: HR = 2.78, PFS: HR = 1.51, *p* < 0.0001) than in LCC (OS: HR = 1.54, PFS: HR = 1.30, *p* < 0.0001). When conducting subgroup analysis by cancer site, no associations were found between FI and 3‐year survival endpoints. HbA1c was significantly associated with poor 3‐year OS for all sites excluding the rectum. HbA1c was associated with poor 3‐year PFS for only LCC (HR = 1.36, 95% CI = 1.01–1.70, *p* < 0.0001). FG‐associated rs7903146 was the only individual SNP that was associated with poor 3‐year PFS in RCC (HR = 4.47, 95% CI = 1.25–15.91, *p* = 0.02 Table S9).

**TABLE 4:** Association between individual SNPs and 3‐year overall survival and progression‐free survival by tumor location (right, left, and rectal) using the multivariate Cox proportional hazards model adjusted for age and sex.

	3‐year survival				5‐year survival			
	All (95% CI)	Right (95% CI)	Left (95% CI)	Rectal (95% CI)	All (95% CI)	Right (95% CI)	Left (95% CI)	Rectal (95% CI)
No. of cases	509	120	234	137	509	120	234	137
*Overall survival*								
No. of events	51	9	22	16	362	79	169	102
Fasting glucose	**1.35 (1.07, 1.63)**	**2.78 (2.03, 3.53)**	**1.54 (1.12, 1.96)**	0.94 (0.41, 1.46)	1.09 (0.99, 1.20)	**1.23 (1.01, 1.46)**	**1.39 (1.24, 1.55)**	**0.74 (0.53, 0.96)**
Fasting insulin^a^	1.37 (0.82, 2.31)	2.27 (0.61, 8.40)	1.58 (0.73, 3.42)	1.06 (0.42, 2.72)	1.06 (0.87, 1.30)	1.00 (0.67, 1.51)	**1.44 (1.08, 1.93)**	0.72 (0.49, 1.08)
HbA1c	**1.38 (1.01, 1.74)**	**2.29 (1.36, 3.21)**	**1.59 (1.04, 2.13)**	1.04 (0.38, 1.71)	1.06 (0.92, 1.20)	1.00 (0.70, 1.29)	**1.44 (1.24, 1.65)**	0.72 (0.44, 1.00)
*Progression‐free survival*								
No. of events	134	28	60	42	375	83	176	104
Fasting glucose	**1.21 (1.04, 1.39)**	**1.51 (1.13, 1.89)**	**1.30 (1.04 1.56)**	1.08 (0.75, 1.40)	1.11 (1.00, 1.21)	1.22 (1.00, 1.44)	**1.39 (1.24, 1.54)**	0.78 (0.57, 1.00)
Fasting insulin^a^	1.19 (0.87, 1.65)	1.19 (0.61, 2.33)	1.36 (0.84 2.21)	1.18 (0.65, 2.13)	1.08 (0.89, 1.32)	0.96 (0.65, 1.44)	**1.44 (1.09, 1.92)**	0.81 (0.54, 1.21)
HbA1c	1.18 (0.96, 1.41)	1.13 (0.65, 1.61)	**1.36 (1.01, 1.70)**	1.13 (0.71, 1.55)	1.07 (0.93, 1.21)	0.95 (0.67, 1.24)	**1.44 (1.24, 1.64)**	0.79 (0.51, 1.07)

Abbreviations: CI, confidence interval; HR, hazard ratio; Hba1c, hemoglobin A1c; SNP, single‐nucleotide polymorphism.

Bolded values were statistically significant (*p* < 0.05) as the values in the parantheses represent the 95% confidence interval.

^a^ Pooled fasting insulin has the same values as the SNP rs17036328 as it was the only SNP available for analysis.

FG was significantly associated with 5‐year OS for each of the three cancer sites. Five‐year OS was poorer in RCC and LCC while it was better in rectal cancer (HR = 0.74, 95% CI = 0.53–0.96, *p* < 0.0001). FI and HbA1c were found to be significantly associated with poor 5‐year OS in LCC. All three glycemic traits were associated with worse 5‐year PFS in LCC only. All SNPs, excluding rs7903146, were associated with poor 5‐year OS in LCC (Table S10). The HR estimates for rs7903146 were significant for both 5‐year OS (HR = 2.49, 95% CI = 1.31–4.37, *p* = 0.005) and 5‐year PFS (HR = 2.73, 95% CI = 1.45–5.13, *p* = 0.001) in RCC only. Schoenfeld residual plots showed no significant correlation between outcomes and time, validating the proportional hazards assumption (Figure S5).

## 4 DISCUSSION

There have been numerous investigations into the impact of metabolic syndrome and diabetes on CRC prognosis, but they have yielded conflicting results.^30^, ^31^ In the present study, we performed a two‐sample MR analysis to examine the potential causal effect of three glycemic traits (FG, FI, HbA1c) on CRC survival. We utilized summary statistics from two GWAS consortiums and genetic data from 509 South Korean CRC patients. Several different MR estimation methods, such as the IVW method, were used to estimate causal effects and we performed sensitivity analyses to validate the MR assumptions. We also conducted a survival analysis using a multivariate Cox proportional hazards model adjusted for age and sex to explore whether cancer staging, and site affected survival outcomes. To the best of our knowledge, no other studies have investigated the potential causal association between glycemic traits and CRC survival utilizing MR in an East Asian population.

Our analysis showed a significant positive causal association between 3‐year OS and HbA1c and validated our results using sensitivity analyses. We did find that the MR–Egger method was more appropriate for analyzing FG and 3‐year OS, as there was evidence of heterogeneity for the IVW method. Our findings suggest that HbA1c may play a protective role in short‐term CRC survival. Although we did calculate the causal OR for each glycemic trait, we reported the causal effect size as our main results as ORs are causal measures more appropriate in a case–control setting^32^ and require additional assumptions on top of the IV assumptions.^33^

Our survival analysis showed that glycemic traits affected CRC survival differently by clinicopathologic traits such as staging and cancer location. We observed that pooled HRs for FG and HbA1c were associated with worse survival for all 3‐year survival endpoints for Stage III. Our results suggest that FG and HbA1c plays an important role in short‐term survival for locally advanced CRC. This finding is consistent with earlier literature that has reported that hyperglycemia increases colon cancer malignancy by promoting cancer cell growth.^34^ We also observed that none of the three glycemic traits were associated with worse Stage II survival. These findings may be explained by the fact that localized Stage II CRC patients generally tend to have high survival rates: the 5‐year survival rate is 80% for Stage II and decreases to 60% for Stage III patients.^35^ We excluded Stage IV patients from our analysis because Stage IV CRC is metastatic, and past studies have excluded Stage IV from their survival analysis or separated their analysis into two groups: localized or metastatic.^36^, ^37^, ^38^ Moreover, diabetic FG levels were associated with an increased risk of death among men with localized but not metastatic prostate cancer.^38^, ^39^ Higher fasting plasma glucose has also been found to be associated with worse OS and PFS in localized Stage III non‐small cell lung cancer.^36^ Studies on the effects of metformin on survival for diabetic patients with CRC have reported stage‐specific results similar to ours; only Stage III patients showed improved survival after taking metformin.^37^, ^40^

Earlier literature has found that diabetes is associated with a higher risk for RCC than LCC,^16^ but little is known about the impact of glycemic traits on CRC survival by cancer site. Our analysis showed similar results: FG and HbA1c were associated with worse 3‐year OS in RCC but not in LCC. We expected LCC to have better survival than RCC, as reported by the Korea Central Cancer Registry.^41^ In contrast to our 3‐year survival results, all three glycemic traits were associated with significantly worse 5‐year survival in LCC. FG was associated with better 5‐year OS in rectal cancer patients, matching findings from a South Korea study that found that diabetic status did not impact survival for rectal cancer.^12^ In summary, our findings suggest that hyperglycemia, and not hyperinsulinemia, plays an important role in CRC progression as FI was not significantly associated with any survival outcomes regardless of cancer location with the exclusion of five‐year survival in LCC. It is unclear why left‐sidedness is associated with worse 5‐year survival, as patients with LCC tend to have better survival, and right‐sidedness has been found be correlated with worse cancer‐specific survival and recurrence‐free survival among South Korean patients.^42^

Earlier MR analyses on the causal effect of T2DM or hyperglycemia on CRC risk and survival have yielded inconsistent results.^5^, ^11^, ^12^, ^43^, ^44^, ^45^ Some studies have reported that no diabetic traits were casually associated with CRC risk^43^, ^45^, ^46^ while IGF1 and IGFBP3 were found to be associated with increased risk in the UK Biobank data.^45^ In terms of CRC survival, diabetes has been strongly associated with higher mortality in meta‐analysis studies, but it is unclear whether common risk factors among diabetic patients (e.g., obesity, smoking, and age) confound this association. Meta‐analyses are also limited in explaining causal associations, as the individual studies used in meta‐analyses have heterogeneous study populations and outcome assessment standards.^47^, ^48^ Furthermore, diabetic status negatively impacts all‐cause mortality rather than specifically cancer‐specific death, thereby making it difficult to determine exactly how diabetic features, such as hyperglycemia and hyperinsulinemia, affect survival.^12^, ^44^

Although the mechanisms are not fully understood, several potential pathways through which FG, FI, and HbA1c may impact CRC survival have been proposed. Hyperglycemia is thought to increase colon cancer malignancy by altering cell glycosylation through the hexosamine biosynthetic pathway.^34^ CRC patients with high blood glucose levels have also been reported to have larger tumor diameters, advanced TNM staging, and ulcerative gross types, but hyperglycemia itself was not found to be associated with distant metastasis or survival.^49^ In localized CRC, Stages I through III, high blood sugar levels (≥110 mg/dL) have been found to be negatively associated with disease‐free survival and OS through the inhibition of miR‐16 expression.^38^ Postoperative fasting blood glucose has also been proposed as a temporal prognostic factor for localized CRC patients with higher postoperative fasting blood glucose leading to better disease‐free survival.^50^ Hyperinsulinemia and changes in the IGF axis have also been suggested as the mechanisms for increased risk of both cancer recurrence and death.^51^ Activation of insulin receptors has been shown to activate mitogenic and antiapoptotic pathways in intestinal epithelial cells and colon cancer cell lines.^52^, ^53^ Insulin also sensitizes cells to growth factors such as IGFs, platelet‐derived growth factor, and vascular endothelial growth factor.^54^ An increase in insulin may also reduce the inhibitory effects of IGFBP‐1 on cancer cell growth and migration.^55^, ^56^

As FG and HbA1c both measure blood glucose concentration, we initially expected them to have similar significant negative causal effects on survival endpoints. It should be noted that discrepancies between FG and HbA1c values have been reported among European and East Asian adult diabetic patients, and the American Diabetes Association (ADA) recommends using HbA1c over FG as a diagnostic marker for diabetes.^57^ HbA1c may be a better measure for prognosis, as it can be tested without fasting and reflects blood glucose levels over the past weeks.^38^ One study found that out of five glycemic traits (fasting blood glucose, insulin, HbA1c, and HOMA‐IR), HbA1c was the only glycemic marker that was significantly associated with CRC progression and was negatively associated with PFS and OS.^44^ Our results were consistent with these previous reports, as HbA1c was the only glycemic trait that was significantly causally associated with 3‐year OS. We did observe that HbA1c had a positive, rather than a negative causal effect on 3‐year OS. The different findings in observational studies and our present MR study leave much to be desired, as there is not a clear answer as to whether and how increased HbA1c influences CRC progression.

The main strength of this study is that it utilized strong IVs that were chosen from established GWAS consortiums such as MAGIC, AGEN, and KBA.^58^ We chose East Asian‐specific SNPs in our analysis which reduces the chances of potential heterogeneity in the causal effects by ethnic differences: MAGIC analyzed individuals of European ancestry, while our study examined a cohort of South Korean patients. Studies using the East Asian‐specific AGEN data have successfully replicated the effect sizes of SNPS in MAGIC for fasting plasma glucose, HbA1c, and insulin resistance.^22^, ^59^ Effect sizes of FG, HbA1c, and insulin resistance SNPs appear to be largely transferable across ethnic groups.^60^ The present study shows that glycemic traits may play an important role in locally advanced CRC prognosis and are not just involved in CRC risk. Previous studies have examined the role of diabetes in CRC risk and survival but have not examined the individual causal effects of diabetes‐associated glycemic traits. By using FG, FI, and HbA1c as our exposure factors, we were able to explore the impact of glycemic traits on CRC survival, independent of diabetic status.

Some limitations should also be considered. Inconsistencies among previous MR studies on CRC risk may have arisen due to the use of weak instrumental variables or the presence of pleiotropy. Although we were able to check for heterogeneity and pleiotropy for FG, we were unable to perform sensitivity analyses for FI as only one variant was available. MR analysis results become stronger when a large number of IVs are utilized, but only a handful of SNPs were available in our study despite using summary statistics from large GWAS. The small sample size of locally advanced CRC cases may also have reduced the statistical power of our MR analysis. Moreover, the validity of any MR study relies on modeling assumptions that must be met for an accurate causal estimate. Two‐sample MR studies are prone to violating certain assumptions, as they cannot be fully tested in the framework of the study design. We also cannot rule out the possibility of confounders, even though MR studies are less susceptible. Detailed information on diabetic status, such as the type (Type 1 or 2), duration of illness, and type of diabetic medication were unavailable in the cohort data. As information regarding the type of diabetes was unavailable, we assumed that patients had T2DM as most Korean adult diabetes (diagnosed age ≥20 years) are T2DM.^61^, ^62^ Diabetes on its own can impact survival through end organ damage but only one patient had severe diabetes that caused end organ damage within the SNUH CRC cohort. We used various sensitivity analyses to ensure that MR assumptions were sufficiently met but the results of the present study should be interpreted with caution. While MR analysis can show the causal relationship between glycemic traits and CRC survival, the exact biological mechanism of such a relationship is yet unknown. One shortcoming of MR analysis is that in using genetic markers that reflect genetically determined exposure traits, MR assumes a constant effect of traits over a lifetime. Glycemic traits can change over time depending on a patient's treatment and this must be considered in interpreting the results of our MR analysis. Another limitation is that a Cox proportional hazard model‐based MR analysis using failure times as outcomes may be biased in their IVW estimates, especially when using summary statistics.^63^, ^64^

To summarize, in this the first MR study that examines the roles of glycemic traits on CRC survival, we found that HbA1c was causally associated with poor OS while there were no significant causal associations between FG and FI and survival. Our results suggest that glycemic traits, regardless of diabetic status, have an impact on CRC survival and that such effects differ by cancer site and location. Glycemic traits such as FG and HbA1c could be used to monitor CRC. Lifestyle and/or pharmacological interventions targeting glycemic traits could also help improve the survival of CRC patients by controlling glycemic levels.

## AUTHOR CONTRIBUTIONS

**So Yon Jun:** Conceptualization (equal); formal analysis (equal); investigation (equal); visualization (equal); writing – original draft (lead); writing – review and editing (equal). **Sooyoung Cho:** Conceptualization (equal); data curation (equal); formal analysis (equal); methodology (equal); visualization (equal); writing – review and editing (equal). **Min Jung Kim:** Data curation (equal); resources (equal); writing – review and editing (equal). **Ji Won Park:** Conceptualization (equal); data curation (equal); funding acquisition (equal); methodology (equal); project administration (equal); resources (equal); supervision (equal); writing – review and editing (equal). **Seung‐Bum Ryoo:** Data curation (equal); resources (equal); writing – review and editing (equal). **Seung Yong Jeong:** Data curation (equal); funding acquisition (equal); resources (equal); writing – review and editing (equal). **Kyu Joo Park:** Data curation (equal); resources (equal); writing – review and editing (equal). **Aesun Shin:** Conceptualization (equal); data curation (equal); funding acquisition (equal); methodology (equal); project administration (equal); resources (equal); supervision (equal); writing – review and editing (equal).

## FUNDING INFORMATION

This work was supported by the National Research Foundation of Korea (NRF) grant funded by the Korean government (MSIT) (No. 2017R1A2B4009233 and 2022R1A2C1004608).

## CONFLICT OF INTEREST STATEMENT

The authors declare no potential conflicts of interest.

## ETHICS STATEMENT

Patients enrolled in the SNUH cohort (IRB No. 1408‐127‐607) all provided written informed consent and the study was approved by the SNUH institutional review board (2017‐11‐09).

## Supporting information


Data S1:

Data S2:

## Data Availability

All data generated or analyzed during this study are included in this published article and its supplementary information files.
