# Supplementary material for: Glycemic traits and colorectal cancer survival in a cohort of South Korean patients: A Mendelian randomization analysis
Source: Cancer Med. 2024 Mar 13;13(5):e7084. doi: 10.1002/cam4.7084 (PMC10935880; doi:10.1002/cam4.7084)
Supplement: Supplementary file 1 — Data S1: [file CAM4-13-e7084-s002.docx]

**Additional file 1: Supplementary Figures and Tables**


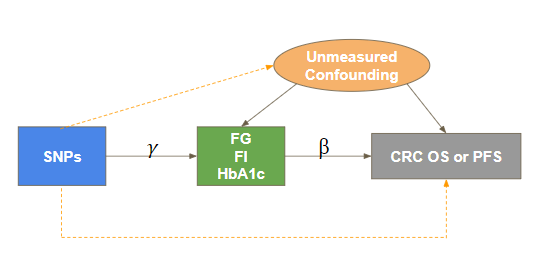


**Figure S1**. Directed acyclic graph (DAG) of our two-sample Mendelian Randomization (MR) study. DAG shows single-nucleotide polymorphisms (SNPs) as instrumental variables, the three glycemic traits as exposure variables, and colorectal cancer overall survival (OS) and progression-free survival (PFS) as outcomes of interest. Dashed lines represent potential pathways that violate MR assumptions

*Abbreviations*: *FG* fasting glucose, *FI* fasting insulin, *HbA1c* hemoglobin A1c


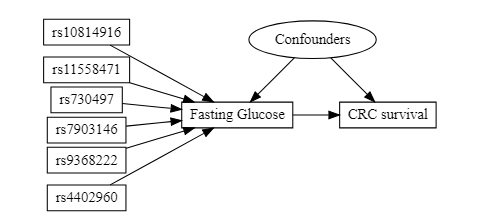


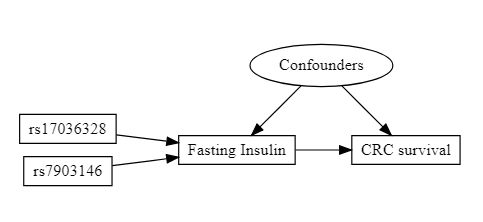


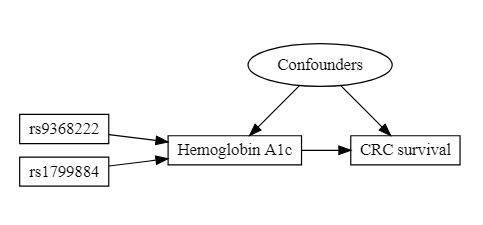


**Figure S2**. Directed acyclic graphs (DAGs) showing genetic variants selected for Mendelian randomization analysis.

(A) Three-year OS (B) Three-Year PFS


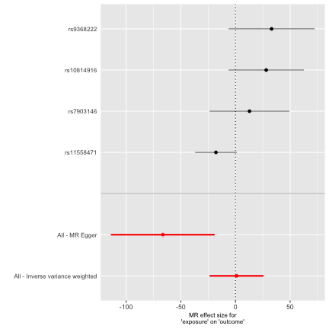

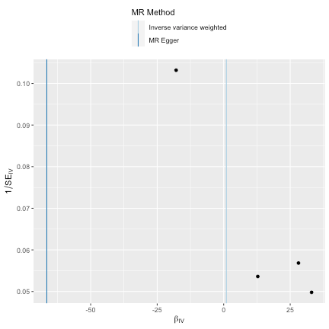

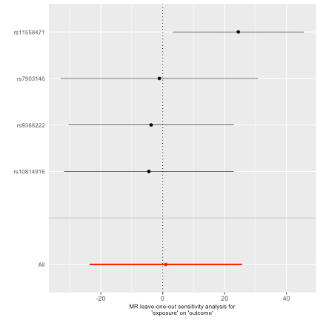

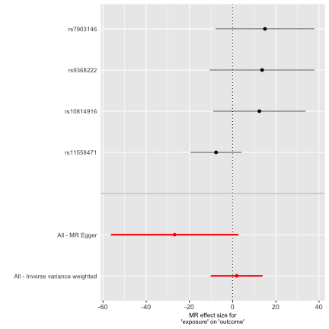

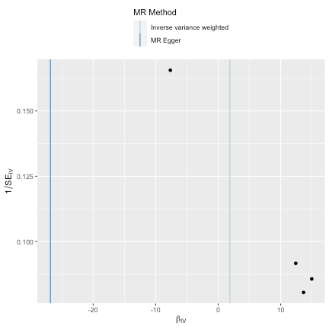

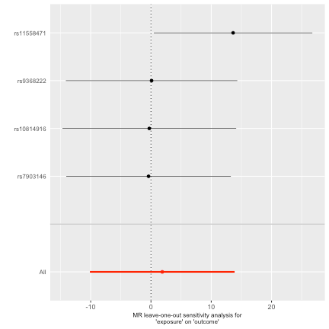


(C) Five-year OS (D) Five-year PFS


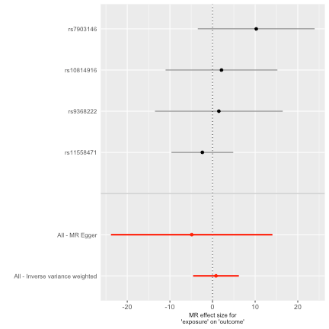

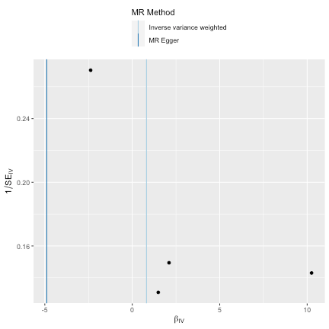

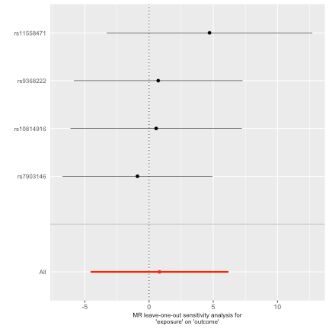

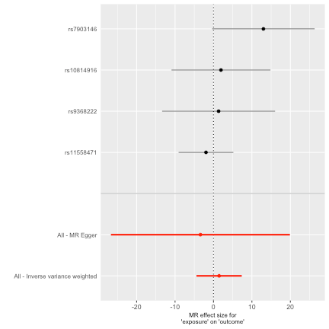

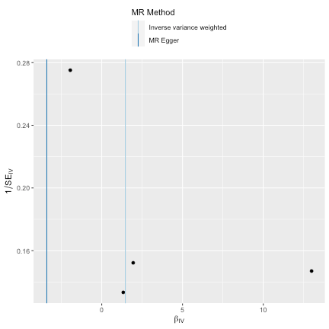

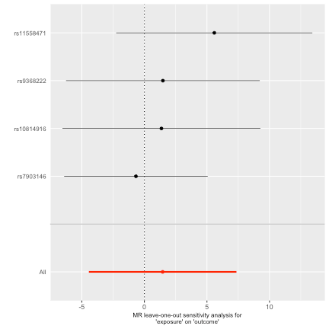


**Figure S3**. Forest plots, funnel plots, and Leave-one-out sensitivity analysis of SNPs for fasting glucose and different survival endpoints (A: three-year OS; B: three-year PFS; C: five-year OS; D: five-year PFS).

*Abbreviations*: *SNPs* single-nucleotide polymorphisms, *OS* overall survival, *PFS* progression-free survival

(A) Three-year OS (B) Three-year PFS


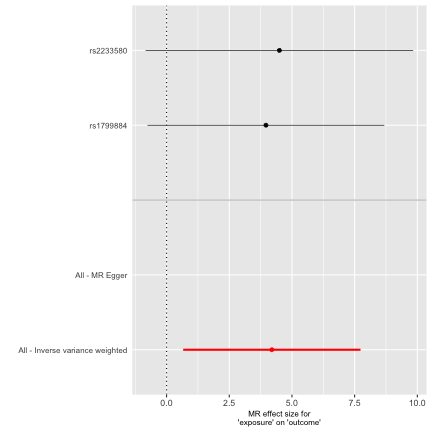

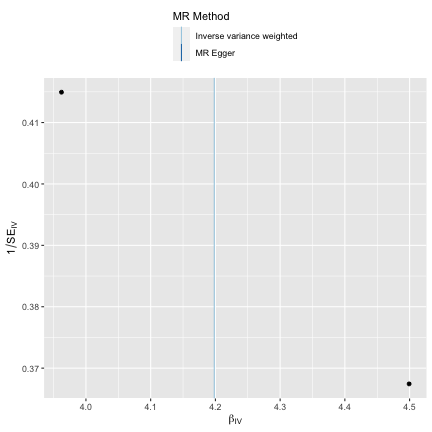

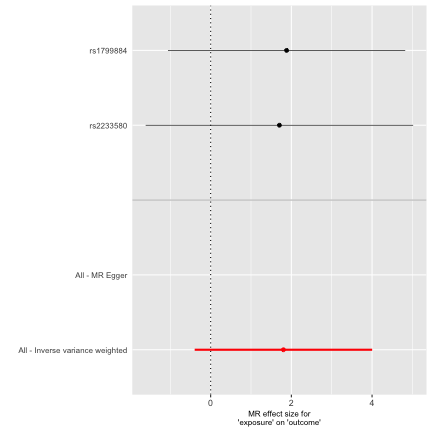

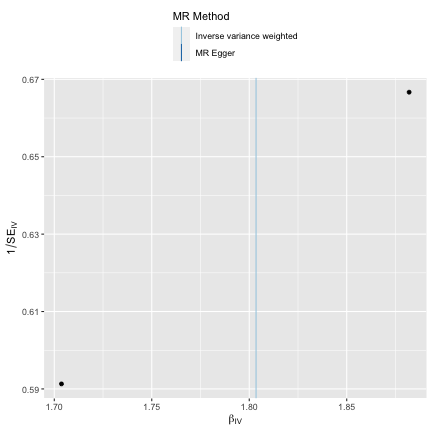


(C) Five-year OS (D) Five-year PFS


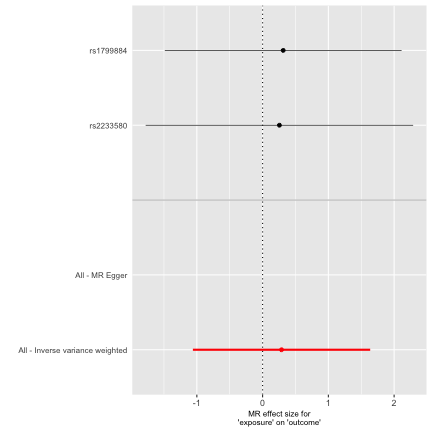

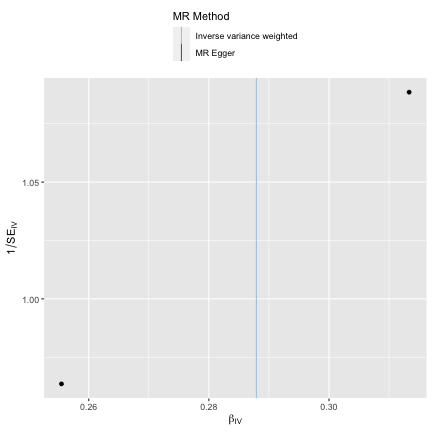

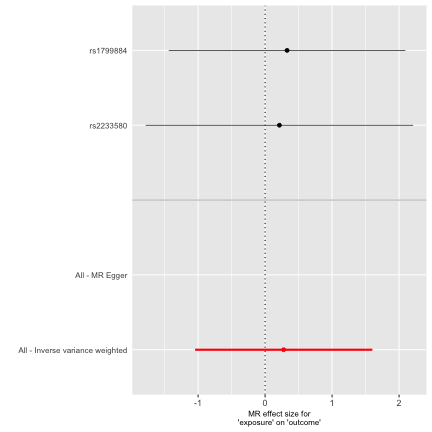

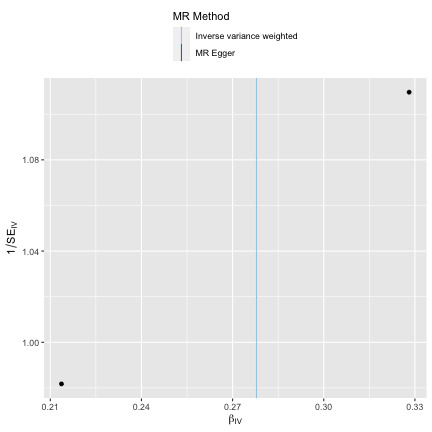


**Figure S4**. Forest plots and funnel plots of SNPs for HbA1c and different survival endpoints (A: three-year OS; B: three-year PFS; C: five-year OS; D: five-year PFS).

*Abbreviations*: *SNPs* single-nucleotide polymorphisms, *HbA1c* hemoglobin A1c, *OS* overall survival, *PFS* progression-free survival

| (A)  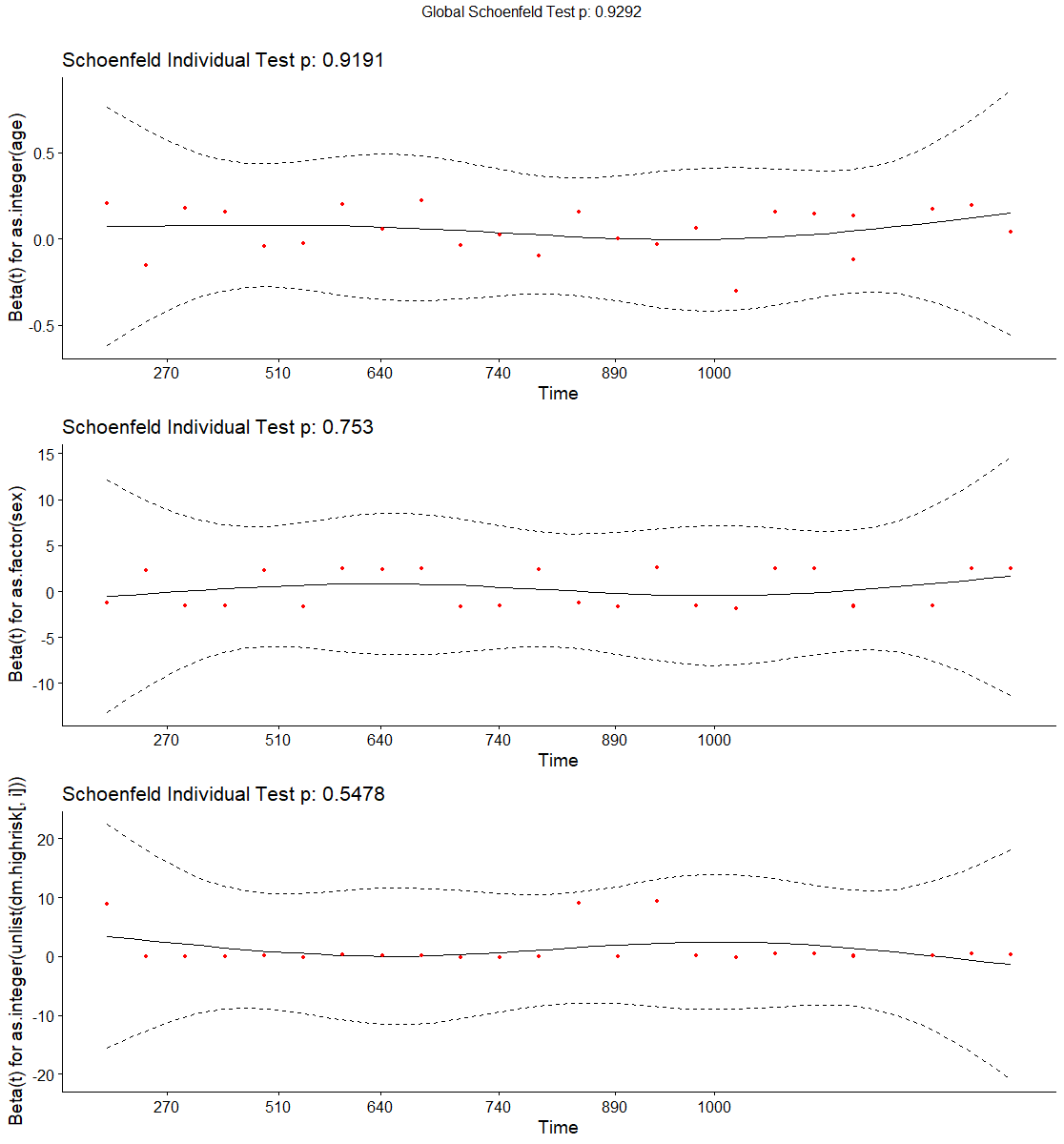 | (B)  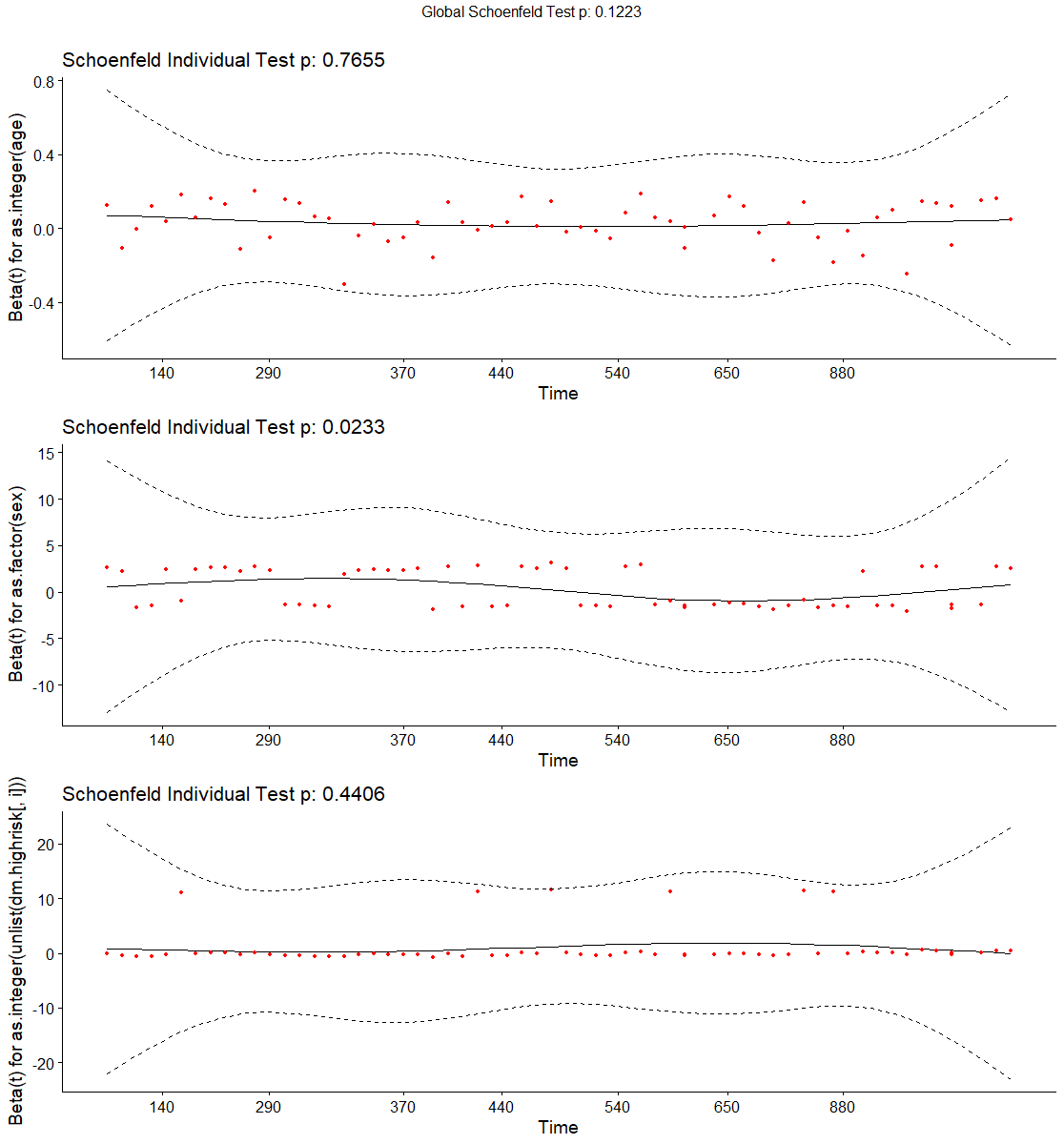 |
| --- | --- |
| (C)  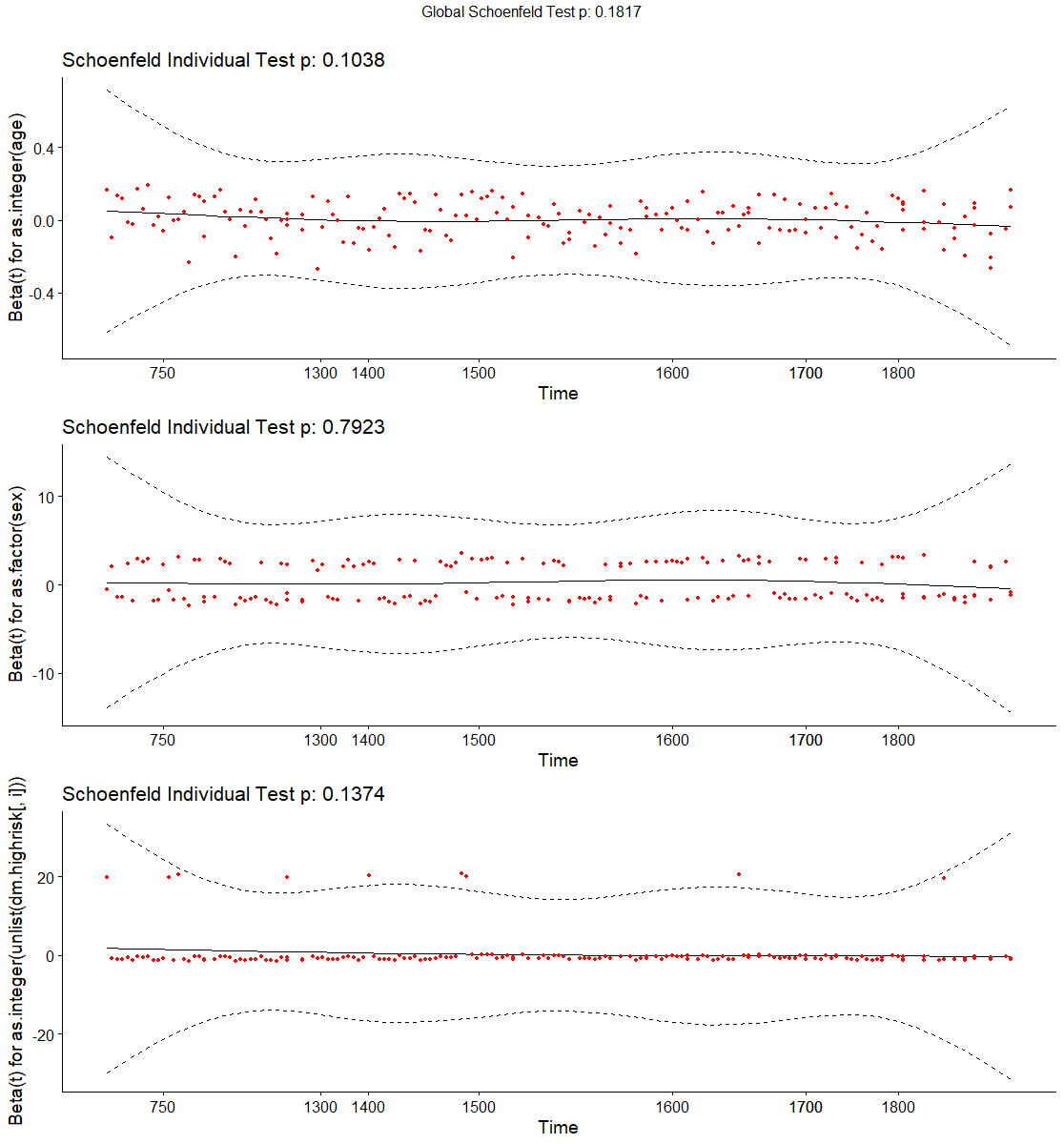 | (D)  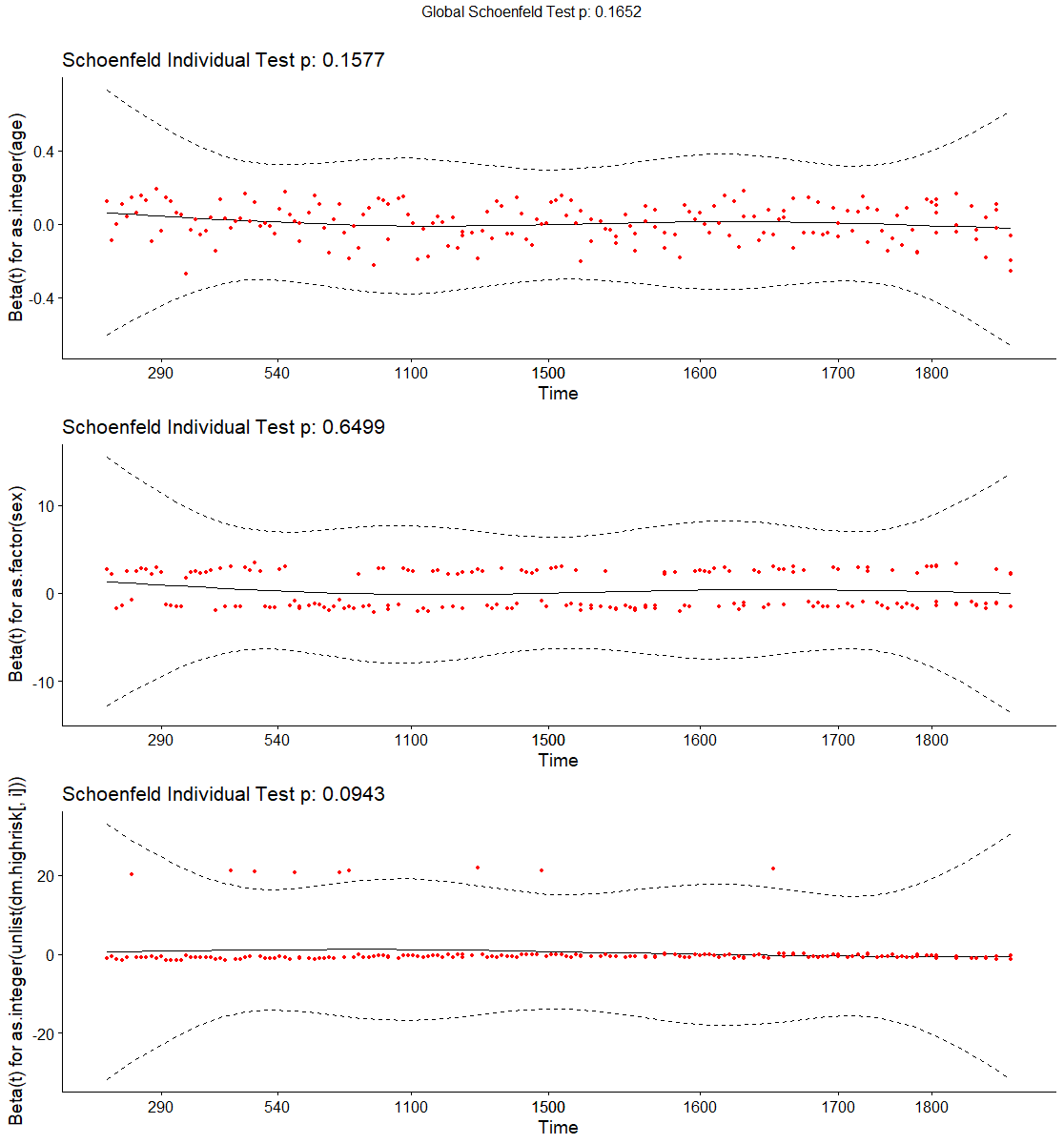 |

**Figure S5**. Schoenfeld residuals for Cox model calculating the casual effect of glycemic traits on survival outcomes for our Mendelian randomization analysis (A: three-year OS; B: three-year PFS; C: five-year OS; D: five-year PFS).

*Abbreviations*: *OS* overall survival, *PFS* progression-free survival

**Table S1.** Baseline characteristics by diabetic status in the SNUH Colorectal Cancer Cohort

| **Characteristics** | **Nondiabetic**  (n=408) | **Diabetic**  (n=101) | **P-value** |
| --- | --- | --- | --- |
| Sex, *n* (%) |  |  | **<0.001** |
| Male | **215 (52.7)** | **74 (73.3)** |  |
| Female | **293 (47.3)** | **27 (26.7)** |  |
| Age, median (IQR) | 60 (53, 70) | 65 (61, 70) | 0.47 |
| BMI, median (IQR) | 23.6 (21.5, 26.0) | 24.2 (22.1, 25.9) | 0.49 |
| Hypertension, *n* (%) | **153 (37.5)** | **62 (61.4)** | **<0.001** |
| Heart Disease, *n* (%) |  |  | 0.18 |
| None | 393 (96.3) | 91 (90.1) |  |
| Ischemic heart disease | 10 (2.5) | 7 (6.9) |  |
| Arrythmia | 4 (1.0) | 1 (1.0) |  |
| Other | 1 (0.2) | 2 (2.0) |  |
| Histologic grade, *n* (%) |  |  | 0.59 |
| 1 | 373 (91.4) | 89 (88.1) |  |
| 2 | 29 (7.1) | 10 (9.9) |  |
| N/A | 6 (1.5) | 2 (2.0) |  |
| Stage, *n* (%) |  |  | 0.62 |
| 2 | 152 (37.3) | 34 (33.7) |  |
| 3 | 256 (62.7) | 67 (66.3) |  |
| Enrollment year, *n* |  |  |  |
| 2014 | 141 | 29 |  |
| 2015 | 174 | 50 |  |
| 2016 | 62 | 15 |  |
| 2017 | 31 | 17 |  |
| Median follow-up, years | 4.65 | 4.61 |  |

*Abbreviations*: *SNUH* Seoul National University Hospital, *n* number, *IQR* interquartile range

**Detailed Information Regarding Mendelian Randomization Analysis:**

We did not find any significant associations between FG and any survival endpoints using other MR methods such as weighted median, simple mode, and weighted mode. (Table S3). To determine which MR method, MR-Egger and IVW, was more valid to estimate the causal effect of FG on three-year OS, we conducted sensitivity analyses. Sensitivity analyses showed that the MR‒Egger method (Q =0.59, *P* =0.75) was a more appropriate estimate than the IVW method (Q =9.12, *P* =0.03) when measuring the causal association between FG and three-year OS. Moreover, the MR‒Egger intercept did not significantly differ from zero (Egger Intercept=1.48, *P*=0.10) (Additional file: supplementary Table 5). No horizontal pleiotropy was directed between FG and other survival endpoints (Tables S5 and S6). Additional sensitivity analyses showed no violations of the IV assumption (Figure S4). LOO analysis showed that no single SNP had a significant influence on the causal estimate for FG on either three-year or five-year survival. The funnel plot showed that the IVW method displayed a slightly more symmetric pattern of effect size variation around the point estimate in comparison to the MR‒Egger method, but the small number of instruments used makes it difficult to accurately assess the symmetry.

HbA1c only had two instruments so LOO analysis was not performed. The funnel plot for HbA1c displayed a symmetrical pattern for all survival points, but it was difficult to assess the validity of the funnel plots (Fig. S4).

**Table S2**. Mendelian randomization analysis results for overall survival and progression-free survival. Wald Ratio estimates for individual SNPs and inverse variance-weighted estimates were calculated to obtain the combined estimate of association for each glycemic trait.

|  | | **3 Year Overall Survival** | | | | **3 Year Progression-free Survival** | | | | **5 Year Overall Survival** | | | | **5 Year Progression-free Survival** | | | | |
| --- | --- | --- | --- | --- | --- | --- | --- | --- | --- | --- | --- | --- | --- | --- | --- | --- | --- | --- |
| **SNP rsID** | | **Wald Ratio**  **β (se)** | ***P*** | **IVW**  **β (se)** | ***P*** | **Wald Ratio**  **β (se)** | ***P*** | **IVW**  **β (se)** | ***P*** | **Wald Ratio**  **β (se)** | ***P*** | **IVW**  **β (se)** | ***P*** | **Wald Ratio**  **β (se)** | ***P*** | **IVW**  **β (se)** | | ***P*** |
| **Fasting Glucose** | | | | | | | |  |  |  |  |  |  |  |  |  |  | |
| rs10814916 | 28.11  (17.79) | | 0.11 | 1.00 (12.57) | 0.94 | 12.41  (10.91) | 0.26 | 1.87  (6.12) | 0.76 | 2.10  (6.69) | 0.75 | 0.81 (2.74) | 0.77 | 1.95  (6.57) | 0.77 | 1.46  (3.01) | 0.63 | |
| rs11558471 | -17.88  (9.69) | | 0.07 |  |  | -7.65  (6.04) | 0.21 |  |  | -2.38  (3.70) | 0.52 |  |  | -1.95  (3.63) | 0.59 |  |  |  |
| rs7903146 | 12.82  (18.65) | | 0.49 |  |  | 14.97  (11.67) | 0.20 |  |  | 10.25  (6.99) | 0.14 |  |  | 12.99  (6.80) | 0.06 |  |  |  |
| rs9368222 | 33.04  (20.08) | | 0.10 |  |  | 13.66  (12.31) | 0.27 |  |  | 1.49  (7.64) | 0.85 |  |  | 1.33  (7.50) | 0.86 |  |  |  |
| **HbA1c** | | | | | | | | | |  |  |  |  |  |  |  |  | |
| rs1799884 | 3.96  (2.41) | | 0.10 | **4.20**  **(1.80)** | **0.02** | 1.18  (1.50) | 0.21 | 1.80  (1.12) | 0.11 | 0.31  (0.92) | 0.73 | 0.29  (0.69) | 0.68 | 0.33  (0.90) | 0.72 | 0.28  (0.67) | 0.68 | |
| rs2233580 | 4.50  (2.72) | | 0.10 |  |  | 1.70  (1.69) | 0.31 |  |  | 0.26  (1.04) | 0.81 |  |  | 0.21  (1.02) | 0.83 |  |  |  |

*Abbreviations*: *SNP* single-nucleotide polymorphism, *IVW* inverse-variance weighted, *HbA1c* hemoglobin A1c

**Table S3**. Other Mendelian randomization methods used to analyze results for overall survival and progression-free survival for fasting glucose.

|  | **3 Year Survival** | | | | | |  | **5 Year Survival** | | | | | |
| --- | --- | --- | --- | --- | --- | --- | --- | --- | --- | --- | --- | --- | --- |
|  | **Weighted median**  **β (se)** | ***P*** | **Simple mode**  **β (se)** | ***P*** | **Weighted mode**  **β (se)** | ***P*** |  | **Weighted median**  **β (se)** | ***P*** | **Simple mode**  **β (se)** | ***P*** | **Weighted mode**  **β (se)** | ***P*** |
| **OS** | 1.56  (9.65) | 0.87 | 27.68 (17.91) | 0.22 | -17.80  (9.64) | 0.16 |  | 0.17  (3.12) | 0.96 | 1.29  (5.13) | 0.82 | -1.84  (3.50) | 0.64 |
| **PFS** | 4.77  (5.54) | 0.39 | 13.68  (12.33) | 0.35 | -7.67  (6.21) | 0.31 |  | 0.22  (3.23) | 0.95 | 1.15  (5.22) | 0.84 | -1.47  (3.43) | 0.70 |

*Abbreviations*: *OS* overall survival, *PFS* progression-free survival

**Table S4**. Odds ratios calculated using Mendelian randomization analysis results for overall survival and progression-free survival.

|  | **3 Year Overall Survival** | | **3 Year Progression-free Survival** | | | | **5 Year Overall Survival** | | **5 Year Progression-free Survival** | | |
| --- | --- | --- | --- | --- | --- | --- | --- | --- | --- | --- | --- |
|  | **IVW OR**  **(95% CI)** | **MR-Egger OR**  **(95% CI)** | | **IVW OR**  **(95% CI)** | **MR-Egger OR**  **(95% CI)** | **IVW OR**  **(95% CI)** | | **MR-Egger OR**  **(95% CI)** | | **IVW OR**  **(95% CI)** | **MR-Egger OR**  **(95% CI)** |
| Fasting Glucose | 2.72  (5.E-11, 1.E+11) | **1.E-29**  **(3.E-50, 5.E-09 )** | | 6.51  (4.E-05, 1.E+06) | 2.E-12  (3.E-25, 1.E+01) | 2.25  (0.01, 487) | | 0.01  (4.E-11, 1.E+06) | | 4.31  (0.01, 2.E+03) | 0.03  (3.E-12, 4.E+08) |
| Fasting Insulin | 4.E-10  (2.E-21, 9.E+01 |  | | 7.E-05  (6.E-12, 8.E+02) |  | 0.20  (9.E-06, 4.E+03) | |  | | 0.14  (8.E-06, 3.E+03) |  |
| HbA1c | 66.57  (1.94, 2.E+03 ) |  | | 6.07  (0.67, 55) |  | 1.33  (0.35, 5) | |  | | 1.32  (0.35, 5) |  |

*Abbreviations*: *CI* confidence interval, *IVW* inverse-variance weighted, *OR* odds ratio, *HbA1c* hemoglobin A1c

**Table S5.** Three-year survival endpoint sensitivity analyses for Mendelian randomization analyses testing for pleiotropy and heterogeneity.

| Heterogeneity | **OS**  **Q (df)** | **P** | **PFS**  **Q (df)** | **P** |
| --- | --- | --- | --- | --- |
| **Fasting Glucose**  MR Egger  Inverse variance weighted | 0.59 (2)  **9.12 (3)** | 0.75  **0.03** | 1.58 (2)  5.58 (3) | 0.45  0.13 |
| **HbA1c**  Inverse variance weighted | 0.02 (1) | 0.88 | 0.01 (1) | 0.94 |
| Pleiotropy | **OS Egger Intercept (se)** | **P** | **PFS egger intercept (se)** | **P** |
| **Fasting Glucose**  Inverse variance weighted | 1.48 (0.51) | 0.10 | 0.63 (0.31) | 0.18 |

*Abbreviations*: *df* degrees of freedom, *OS* overall survival, *PFS* progression free survival, *Q* Cochran’s Q, *HbA1c* hemoglobin A1c

**Table S6.** Five-year survival endpoint sensitivity analyses for Mendelian randomization analyses testing for pleiotropy and heterogeneity.

| Heterogeneity | **OS**  **Q (df)** | **P** | **PFS**  **Q (df)** | **P** |
| --- | --- | --- | --- | --- |
| **Fasting Glucose**  MR Egger  Inverse variance weighted | 2.19 (2)  2.61 (3) | 0.33  0.46 | 3.44 (2)  3.76 (3) | 0.18  0.29 |
| **HbA1c**  Inverse variance weighted | 0.00 (1) | 0.97 | 0.01 (1) | 0.93 |
| Pleiotropy | **OS Egger Intercept (se)** | **P** | **PFS egger intercept (se)** | **P** |
| **Fasting Glucose**  Inverse variance weighted | 0.12 (0.20) | 0.60 | 0.11 (0.25) | 0.71 |

*Abbreviations*: *df* degrees of freedom, *OS* overall survival, *PFS* progression free survival, *Q* Cochran’s Q, *HbA1c* hemoglobin A1c

|  | **3 Year Overall Survival** | | | **3 Year Progression-free Survival** | | |
| --- | --- | --- | --- | --- | --- | --- |
| **SNP rsID** | **All**  **(95% CI)** | **Stage** **II**  **(95% CI)** | **Stage III**  **(95% CI)** | **All**  **(95% CI)** | **Stage** **II**  **(95% CI)** | **Stage III**  **(95% CI)** |
| *No. of cases*  *No. of events* | 509  51 | 186  12 | 323  39 | 509  134 | 186  93 | 323  244 |
| rs17036328 | 1.58  (0.91, 2.73) | 0.43  (0.11, 1.59) | **2.26**  **(1.18, 4.31)** | 1.22  (0.87, 1.72) | 0.81  (0.43, 1.52) | 1.46  (0.97, 2.20) |
| rs9368222 | 1.59  (0.92, 2.76) | 0.43  (0.12, 1.61) | **2.28**  **(1.19, 4.35)** | 1.21  (0.86, 1.70) | 0.78  (0.41, 1.47) | 1.47  (0.97, 2.20) |
| rs1799884 | 1.59  (0.92, 2.75) | 0.45  (0.12, 1.65) | **2.21**  **(1.16, 4.21)** | 1.25  (0.88, 1.75) | 0.82  (0.43, 1.56) | 1.50  (0.99, 2.26) |
| rs2233580 | 1.59  (0.92, 2.76) | 0.44  (0.12, 1.64) | **2.24**  **(1.18, 4.27)** | 1.19  (0.85, 1.68) | 0.75  (0.39, 1.43) | 1.45  (0.97, 2.18) |
| rs11558471 | 1.68  (0.97, 2.91) | 0.43  (0.12, 1.64) | **2.42**  **(1.27, 4.62)** | 1.25  (0.89, 1.76) | 0.85  (0.44, 1.61) | 1.48  (0.98, 2.22) |
| rs10814916 | 1.57  (0.90, 2.72) | 0.42  (0.11, 1.58) | **2.24**  **(1.17, 4.27)** | 1.22  (0.87, 1.72) | 0.81  (0.43, 1.53) | 1.46  (0.97, 2.20) |
| rs7903146 | 1.33  (0.59, 2.96) | 0.00  (0.00, inf) | 2.41  (0.95, 6.16) | 1.39  (0.84, 2.30) | 0.87  (0.37, 2.06) | 1.80  (0.96, 3.39) |

**Table S7**. Association between individual SNPs and three-year overall survival and progression-free survival by cancer stage using multivariate Cox proportional hazard model adjusted for age and sex. Bolded numbers represent significant values.

*Abbreviations*: *SNP* single-nucleotide polymorphism, *CI* confidence interval, *HR* hazard ratio

|  | **5 Year Overall Survival** | | | **5 Year Progression-Free Survival** | | |
| --- | --- | --- | --- | --- | --- | --- |
| **SNP rsID** | **All**  **(95% CI)** | **Stage** **II**  **(95% CI)** | **Stage III**  **(95% CI)** | **All**  **(95% CI)** | **Stage** **II**  **(95% CI)** | **Stage III**  **(95% CI)** |
| *No. of cases*  *No. of events* | 509  362 | 186  129 | 323  233 | 509  375 | 186  131 | 323  244 |
| rs17036328 | 1.03  (0.84, 1.28) | 0.97  (0.68, 1.38) | 1.07  (0.82, 1.39) | 1.04  (0.85, 1.28) | 0.95  (0.67, 1.36) | 1.09  (0.85, 1.41) |
| rs9368222 | 1.02  (0.83, 1.26) | 0.93  (0.65, 1.32) | 1.07  (0.83, 1.39) | 1.02  (0.83, 1.25) | 0.90  (0.63, 1.28) | 1.09  (0.84, 1.40) |
| rs1799884 | 1.04  (0.84, 1.28) | 0.98  (0.69, 1.40) | 1.06  (0.81, 1.37) | 1.04  (0.85, 1.28) | 0.96  (0.67, 1.36) | 1.08  (0.84, 1.40) |
| rs2233580 | 1.03  (0.83, 1.27) | 0.95  (0.67, 1.36) | 1.06  (0.82, 1.38) | 1.02  (0.83, 1.26) | 0.93  (0.65, 1.32) | 1.08  (0.83, 1.39) |
| rs11558471 | 1.07  (0.87, 1.32) | 0.95  (0.67, 1.36) | 1.14  (0.88, 1.48) | 1.06  (0.86, 1.30) | 0.95  (0.66, 1.35) | 1.13  (0.87, 1.45) |
| rs10814916 | 1.03  (0.84, 1.28) | 0.96  (0.67, 1.37) | 1.08  (0.83, 1.40) | 1.03  (0.84, 1.27) | 0.94  (0.66, 1.34) | 1.09  (0.84, 1.41) |
| rs7903146 | 1.25  (0.93, 1.69) | 0.97  (0.59, 1.59) | 1.37  (0.94, 2.02) | 1.33  (0.99, 1.78) | 1.03  (0.63, 1.69) | **1.52**  **(1.05, 2.19)** |

**Table S8**. Association between individual SNPs and five-year overall survival and progression-free survival by cancer stage using multivariate Cox proportional hazard model adjusted for age and sex. Bolded numbers represent significant values.

*Abbreviations*: *SNP* single-nucleotide polymorphism, *CI* confidence interval, *HR* hazard ratio

**Table S9**. Association between individual SNPs and three-year overall survival and progression-free survival by tumor location (right, left, rectal) using multivariate Cox proportional hazard model adjusted for age and sex. Bolded numbers represent significant values.

|  | **3 Year Overall Survival** | | | | **3 Year Progression-Free Survival** | | | |
| --- | --- | --- | --- | --- | --- | --- | --- | --- |
| **SNP rsID** | **All**  **(95% CI)** | **Right**  **(95% CI)** | **Left**  **(95% CI)** | **Rectal**  **(95% CI)** | **All**  **(95% CI)** | **Right**  **(95% CI)** | **Left**  **(95% CI)** | **Rectal**  **(95% CI)** |
| *No. of cases*  *No. of events* | 509  51 | 120  9 | 234  22 | 137  16 | 509  134 | 120  28 | 234  60 | 137  42 |
| rs17036328 | 1.37  (0.82, 2.31) | 2.27  (0.61, 8.40) | 1.58  (0.73, 3.42) | 1.06  (0.42, 2.72) | 1.19  (0.87, 1.65) | 1.19  (0.61, 2.33) | 1.36  (0.84 2.21) | 1.18  (0.65, 2.13) |
| rs9368222 | 1.35  (0.81, 2.28) | 2.24  (0.60, 8.36) | 1.53  (0.71, 3.30) | 1.06  (0.41, 2.72) | 1.17  (0.85, 1.61) | 1.17  (0.59, 2.29) | 1.34  (0.82, 2.17) | 1.12  (0.62, 2.03) |
| rs1799884 | 1.35  (0.81, 2.28) | 2.03  (0.54, 7.56) | 1.60  (0.74, 3.48) | 1.04  (0.40, 2.71) | 1.21  (0.88, 1.68) | 1.13  (0.57, 2.23) | 1.46  (0.89, 2.40) | 1.07  (0.58, 1.96) |
| rs2233580 | 1.38  (0.82, 2.32) | 2.31  (0.62, 8.56) | 1.59  (0.73, 3.45) | 1.03  (0.40, 2.63) | 1.17  (0.85, 1.62) | 1.07  (0.54, 2.12) | 1.36  (0.83, 2.21) | 1.08  (0.59, 1.97) |
| rs11558471 | 1.45  (0.86, 2.45) | 3.76  (0.81, 17.51) | 1.58  (0.73, 3.48) | 0.98  (0.38, 2.52) | 1.23  (0.89, 1.70) | 1.28  (0.62, 2.44) | 1.37  (0.84, 2.22) | 1.11  (0.60, 2.06) |
| rs10814916 | 1.31  (0.78, 2.21**)** | 2.14  (0.58, 7.93) | 1.60  (0.73, 3.48) | 0.94  (0.36, 2.45) | 1.18  (0.85, 1.62) | 1.23  (0.62, 2.44) | 1.32  (0.81, 2.15) | 1.10  (0.60, 2.00) |
| rs7903146 | 1.19  (0.52, 2.70) | 4.09  (0.46, 36.65) | 1.34  (0.39, 4.64) | 0.26  (0.04, 1.69) | 1.36  (0.84, 2.19) | **4.47**  **(1.25, 15.91)** | 1.06  (0.52, 2.16) | 0.86  (0.35, 2.15) |

*Abbreviations*: *SNP* single-nucleotide polymorphism, *CI* confidence interval, *HR* hazard ratio

**Table S10**. Association between individual SNPs and five-year overall survival and progression-free survival by tumor location (right, left, rectal) using multivariate Cox proportional hazard model adjusted for age and sex. Bolded numbers represent significant values.

*Abbreviations*: *SNP* single-nucleotide polymorphism, *CI* confidence interval, *HR* hazard ratio

|  | **5 Year Overall Survival** | | | | **5 Year Progression-free Survival** | | | |
| --- | --- | --- | --- | --- | --- | --- | --- | --- |
| **SNP rsID** | **All**  **(95% CI)** | **Right**  **(95% CI)** | **Left**  **(95% CI)** | **Rectal**  **(95% CI)** | **All**  **(95% CI)** | **Right**  **(95% CI)** | **Left**  **(95% CI)** | **Rectal**  **(95% CI)** |
| *No. of cases*  *No. of events* | 509  362 | 120  79 | 234  169 | 137  102 | 509  375 | 120  83 | 234  176 | 137  104 |
| rs17036328 | 1.06  (0.87, 1.30) | 1.00  (0.67, 1.51) | **1.44**  **(1.08, 1.93)** | 0.72  (0.49, 1.08) | 1.08  (0.89, 1.32) | 0.96  (0.65, 1.44) | 1.44  (1.09, 1.92) | 0.81  (0.54, 1.21) |
| rs9368222 | 1.05  (0.86, 1.27) | 1.03  (0.68, 1.55) | **1.40**  **(1.05, 1.87)** | 0.76  (0.51, 1.13) | 1.05  (0.87, 1.28) | 0.98  (0.65, 1.46) | 1.39  (1.04, 1.84) | 0.82  (0.55, 1.22) |
| rs1799884 | 1.07  (0.88, 1.30) | 1.00  (0.66, 1.51) | **1.48**  **(1.10, 1.97)** | 0.77  (0.51, 1.14) | 1.08  (0.89, 1.31) | 0.95  (0.64, 1.42) | 1.49  (1.12, 1.99) | 0.82  (0.55, 1.22) |
| rs2233580 | 1.05  (0.86, 1.29) | 0.99  (0.65, 1.49) | **1.44**  **(1.08, 1.93)** | 0.72  (0.49, 1.07) | 1.06  (0.87, 1.29) | 0.94  (0.63, 1.41) | 1.44  (1.08, 1.92) | 0.77  (0.52, 1.14) |
| rs11558471 | 1.11  (0.91, 1.35) | 1.11  (0.73, 1.68) | **1.45**  **(1.09, 1.94)** | 0.75  (0.51, 1.12) | 1.10  (0.91, 1.34) | 1.05  (0.70, 1.58) | 1.45  (1.09, 1.92) | 0.77  (0.52, 1.15) |
| rs10814916 | 1.06  (0.87, 1.30) | 1.03  (0.68, 1.57) | **1.44**  **(1.08, 1.93)** | 0.73  (0.49, 1.08) | 1.07  (0.88, 1.30) | 1.00  (0.67, 1.50) | 1.42  (1.07, 1.89) | 0.78  (0.53, 1.15) |
| rs7903146 | 1.23  (0.92, 1.64) | **2.49**  **(1.31, 4.73)** | 1.15  (0.77, 1.73) | 0.70  (0.38, 1.26) | 1.32  (1.00, 1.75) | **2.73**  **(1.45, 5.13)** | 1.21  (0.82, 1.80) | 0.74  (0.41, 1.34) |
