# Supplementary material for: Glycemic traits and colorectal cancer survival in a cohort of South Korean patients: A Mendelian randomization analysis
Source: Cancer Med. 2024 Mar 13;13(5):e7084. doi: 10.1002/cam4.7084 (PMC10935880; doi:10.1002/cam4.7084)
Supplement: Supplementary file 2 — Data S2: [file CAM4-13-e7084-s001.docx]

**Additional file 2: SNP Information**

**Table 1.** Statistically significant genetic instruments for all three glycemic traits obtained from the Meta-Analyses of Glucose and Insulin-related traits Consortium (MAGIC).

| **Trait** | **rsID** | **Chr** | **Effect Allele** | **Other Allele** | **Effect size (β)** | **SE** | **P-value** | **EAF** |
| --- | --- | --- | --- | --- | --- | --- | --- | --- |
| FG | rs10830963 | NA | G | C | -0.079 | 0.0037 | 2.20E-100 | NA |
| FG | rs11558471 | NA | G | A | 0.029 | 0.0034 | 7.07E-18 | NA |
| FG | rs11603334 | NA | G | A | -0.023 | 0.0041 | 2.92E-08 | NA |
| FG | rs2191349 | NA | G | T | 0.028 | 0.0031 | 1.98E-20 | NA |
| FG | rs3770568 | NA | C | A | 0.042 | 0.0053 | 3.62E-15 | NA |
| FG | rs4869272 | NA | C | T | 0.02 | 0.0033 | 2.70E-09 | NA |
| FG | rs560887 | NA | C | T | -0.071 | 0.0034 | 2.63E-99 | NA |
| FG | rs6113722 | NA | G | A | -0.042 | 0.0077 | 3.89E-08 | NA |
| FG | rs758989 | NA | C | T | 0.024 | 0.0031 | 3.58E-15 | NA |
| FG | rs983309 | NA | G | T | 0.03 | 0.0048 | 7.61E-10 | NA |
| FG | rs1260326 | NA | T | C | 0.029 | 0.0021 | 2.17E-41 | NA |
| FG | rs1280 | NA | T | C | -0.026 | 0.0031 | 8.56E-18 | NA |
| FG | rs7651090 | NA | A | G | 0.013 | 0.0023 | 1.75E-08 | NA |
| FG | rs11715915 | NA | C | T | -0.012 | 0.0022 | 4.90E-08 | NA |
| FG | rs9368222 | NA | C | A | 0.014 | 0.0023 | 1.00E-09 | NA |
| FG | rs6975024 | NA | T | C | 0.061 | 0.0029 | 2.88E-99 | NA |
| FG | rs17168486 | NA | C | T | 0.031 | 0.0028 | 3.17E-28 | NA |
| FG | rs3829109 | NA | G | A | -0.017 | 0.0027 | 1.13E-10 | NA |
| FG | rs10814916 | NA | A | C | 0.016 | 0.0022 | 2.26E-13 | NA |
| FG | rs10811661 | NA | T | C | -0.024 | 0.0028 | 5.65E-18 | NA |
| FG | rs7903146 | NA | C | T | 0.022 | 0.0024 | 2.71E-20 | NA |
| FG | rs11607883 | NA | G | A | -0.021 | 0.0021 | 6.32E-24 | NA |
| FG | rs174576 | NA | C | A | -0.02 | 0.0022 | 1.18E-18 | NA |
| FG | rs11619319 | NA | A | G | 0.02 | 0.0024 | 1.33E-15 | NA |
| FG | rs12888855 | NA | C | A | -0.016 | 0.0025 | 5.04E-10 | NA |
| FG | rs4502156 | NA | T | C | -0.022 | 0.0021 | 1.38E-25 | NA |
| FG | rs11210771 | NA | T | C | 0.6385 | 0.0291 | 0.0045 | NA |
| FG | rs12712928 | NA | G | C | 0.3741 | 0.0776 | 0.0045 | NA |
| FG | rs1912980 | NA | C | T | 0.3358 | NA | 0.0045 | NA |
| FG | rs3813814 | NA | G | A | 0.4308 | NA | 0.0043 | NA |
| FG | rs832189 | NA | T | C | 0.6387 | 0.0316 | 0.0045 | NA |
| FG | rs1250123 | NA | A | G | 0.5721 | 0.0261 | 0.0043 | NA |
| FG | rs9379084 | NA | G | A | 0.1622 | NA | 0.0058 | NA |
| FG | rs742761 | NA | C | T | 0.2108 | NA | 0.0053 | NA |
| FG | rs58117295 | NA | T | C | 0.17 | 0.0735 | 0.0058 | NA |
| FG | rs9411372 | NA | G | A | 0.256 | 0.0335 | 0.0049 | NA |
| FG | rs10882288 | NA | C | T | 0.4543 | NA | 0.0043 | NA |
| FG | rs60808706 | NA | G | A | 0.3925 | NA | 0.0045 | NA |
| FG | rs671 | NA | G | A | 0.1546 | NA | 0.0059 | NA |
| FG | rs4992759 | NA | T | C | 0.8227 | NA | 0.0057 | NA |
| FG | rs34238147 | NA | G | A | 0.4489 | 0.0301 | 0.0043 | NA |
| FG | rs4965430 | NA | C | G | 0.4866 | NA | 0.0044 | NA |
| FG | rs7219123 | NA | G | C | 0.4472 | NA | 0.0043 | NA |
| FG | rs2302783 | NA | T | C | 0.5489 | NA | 0.0043 | NA |
| FG | rs1964272 | NA | G | A | 0.3702 | NA | 0.0044 | NA |
| FG | rs1757697 | NA | A | G | 0.3011 | NA | 0.0047 | NA |
| FI | rs4846565 | NA | A | G | -0.013 | 0.0022 | 1.76E-09 | NA |
| FI | rs1260326 | NA | C | T | 0.021 | 0.0021 | 2.74E-22 | NA |
| FI | rs10195252 | NA | C | T | -0.017 | 0.0021 | 1.26E-16 | NA |
| FI | rs2943645 | NA | T | C | 0.019 | 0.0021 | 2.26E-19 | NA |
| FI | rs17036328 | NA | C | T | -0.021 | 0.003 | 3.59E-12 | NA |
| FI | rs3822072 | NA | A | G | 0.012 | 0.0021 | 1.80E-08 | NA |
| FI | rs974801 | NA | G | A | 0.014 | 0.0021 | 3.27E-11 | NA |
| FI | rs4865796 | NA | A | G | 0.015 | 0.0022 | 2.16E-12 | NA |
| FI | rs6912327 | NA | C | T | -0.016 | 0.0029 | 2.26E-08 | NA |
| FI | rs2126259 | NA | C | T | -0.024 | 0.0033 | 3.30E-13 | NA |
| FI | rs731839 | NA | A | G | -0.015 | 0.0021 | 5.13E-12 | NA |
| FI | rs2114912 | NA | T | G | 0.026 | 0.0041 | 3.72E-10 | NA |
| HbA1c | rs10823343 | 10 | A | G | 0.0325 | 0.002296 | 2.00E-55 | 0.7223 |
| HbA1c | rs10830963 | 11 | G | C | 0.0196 | 0.002041 | 2.00E-23 | 0.2938 |
| HbA1c | rs11248914 | 16 | T | C | 0.0142 | 0.002041 | 3.00E-14 | 0.647 |
| HbA1c | rs11558471 | 8 | A | G | 0.0213 | 0.003827 | 1.00E-08 | 0.5391 |
| HbA1c | rs11603334 | 11 | G | A | 0.012 | 0.002092 | 7.00E-09 | 0.815 |
| HbA1c | rs11964178 | 6 | A | G | 0.0096 | 0.001582 | 6.00E-10 | 0.5666 |
| HbA1c | rs12132919 | 1 | A | C | -0.0289 | 0.004592 | 3.00E-11 | 0.2803 |
| HbA1c | rs12621844 | 2 | T | C | 0.0099 | 0.001786 | 2.00E-08 | 0.5999 |
| HbA1c | rs13134327 | 4 | A | G | 0.0131 | 0.001684 | 3.00E-15 | 0.3335 |
| HbA1c | rs1558902 | 16 | A | T | 0.0103 | 0.001888 | 3.00E-08 | 0.4128 |
| HbA1c | rs17509001 | 2 | C | T | 0.0181 | 0.002296 | 2.00E-15 | 0.1576 |
| HbA1c | rs17533903 | 19 | A | G | 0.015 | 0.002041 | 5.00E-12 | 0.2428 |
| HbA1c | rs17747324 | 10 | C | T | 0.0149 | 0.002296 | 6.00E-11 | 0.2489 |
| HbA1c | rs198846 | 6 | G | NA | 0.0218 | 0.002296 | 1.00E-23 | 0.8293 |
| HbA1c | rs2383208 | 9 | A | G | 0.0142 | 0.002041 | 7.00E-12 | 0.7992 |
| HbA1c | rs282587 | 13 | G | NA | 0.019 | 0.002551 | 2.00E-12 | 0.1513 |
| HbA1c | rs3782123 | 11 | C | A | 0.0126 | 0.00199 | 2.00E-10 | 0.3205 |
| HbA1c | rs4607517 | 7 | A | NA | 0.0306 | 0.002296 | 9.00E-38 | 0.2017 |
| HbA1c | rs4737009 | 8 | A | G | 0.0212 | 0.002041 | 4.00E-27 | 0.2531 |
| HbA1c | rs4820268 | 22 | G | NA | 0.0162 | 0.001786 | 1.00E-22 | 0.4606 |
| HbA1c | rs560887 | 2 | C | T | 0.0284 | 0.001786 | 1.00E-58 | 0.6843 |
| HbA1c | rs579459 | 9 | C | T | 0.0107 | 0.001888 | 9.00E-09 | 0.2389 |
| HbA1c | rs592423 | 6 | A | C | 0.0091 | 0.001684 | 4.00E-08 | 0.4566 |
| HbA1c | rs6980507 | 8 | A | G | 0.0097 | 0.001786 | 4.00E-08 | 0.4014 |
| HbA1c | rs7616006 | 3 | A | G | 0.0103 | 0.001684 | 5.00E-10 | 0.5744 |
| HbA1c | rs8192675 | 3 | T | C | 0.0112 | 0.001684 | 1.00E-11 | 0.6906 |
| HbA1c | rs857691 | 1 | T | C | 0.0193 | 0.001786 | 4.00E-25 | 0.2715 |
| HbA1c | rs9818758 | 3 | A | NA | 0.0121 | 0.00199 | 8.00E-10 | 0.2028 |
| HbA1c | rs9914988 | 17 | A | G | 0.0131 | 0.00199 | 3.00E-11 | 0.7877 |
| HbA1c | rs3093638 | 1 | A | G | -0.0529 | 0.0066 | 1.19E-15 | 0.6653 |
| HbA1c | rs72898974 | 1 | T | C | -0.0612 | 0.0102 | 1.93E-09 | 0.1047 |
| HbA1c | rs60724735 | 1 | C | T | -0.1019 | 0.0074 | 1.18E-43 | 0.2333 |
| HbA1c | rs2251963 | 1 | A | G | 0.0621 | 0.0063 | 9.02E-23 | 0.4154 |
| HbA1c | rs340515 | 2 | T | G | -0.0655 | 0.0065 | 4.79E-24 | 0.6153 |
| HbA1c | rs12053049 | 2 | C | T | 0.0712 | 0.0066 | 3.15E-27 | 0.3538 |
| HbA1c | rs1045661 | 2 | C | G | 0.0712 | 0.0074 | 4.42E-22 | 0.2349 |
| HbA1c | rs507131 | 3 | A | C | -0.0496 | 0.0064 | 8.09E-15 | 0.397 |
| HbA1c | rs11569142 | 4 | T | G | -0.0496 | 0.0073 | 1.01E-11 | 0.2482 |
| HbA1c | rs4565031 | 4 | A | G | 0.0376 | 0.0063 | 2.11E-09 | 0.5115 |
| HbA1c | rs10440833 | 6 | A | T | 0.0644 | 0.0063 | 1.44E-24 | 0.4618 |
| HbA1c | rs198851 | 6 | G | T | 0.0982 | 0.0152 | 1.06E-10 | 0.9565 |
| HbA1c | rs34046799 | 6 | A | C | 0.0704 | 0.0097 | 2.92E-13 | 0.1181 |
| HbA1c | rs742761 | 6 | T | C | -0.0515 | 0.0077 | 2.53E-11 | 0.2108 |
| HbA1c | rs9376090 | 6 | C | T | -0.0838 | 0.0067 | 3.09E-36 | 0.3252 |
| HbA1c | rs1799884 | 7 | T | C | 0.1166 | 0.0081 | 1.66E-47 | 0.1822 |
| HbA1c | rs111754661 | 7 | C | T | -0.0592 | 0.01 | 3.38E-09 | 0.1101 |
| HbA1c | rs2233580 | 7 | T | C | 0.1031 | 0.0124 | 9.27E-17 | 0.0686 |
| HbA1c | rs4737009 | 8 | A | G | 0.0698 | 0.0062 | 4.31E-29 | 0.5199 |
| HbA1c | rs34432089 | 8 | A | G | -0.047 | 0.0064 | 3.13E-13 | 0.3872 |
| HbA1c | rs13266634 | 8 | T | C | -0.059 | 0.0064 | 2.61E-20 | 0.3956 |
| HbA1c | rs10965248 | 9 | C | T | -0.0594 | 0.0063 | 3.94E-21 | 0.4375 |
| HbA1c | rs28376933 | 9 | G | A | 0.0666 | 0.0104 | 1.37E-10 | 0.1046 |
| HbA1c | rs550057 | 9 | T | C | 0.0668 | 0.0071 | 5.55E-21 | 0.2591 |
| HbA1c | rs61848342 | 10 | C | T | 0.0442 | 0.0063 | 1.60E-12 | 0.4772 |
| HbA1c | rs2305196 | 10 | A | G | -0.0374 | 0.0063 | 3.07E-09 | 0.5152 |
| HbA1c | rs77329181 | 11 | T | C | 0.0529 | 0.0069 | 2.66E-14 | 0.5262 |
| HbA1c | rs2237897 | 11 | T | C | -0.058 | 0.0068 | 2.04E-17 | 0.3925 |
| HbA1c | rs1002226 | 11 | T | C | -0.0481 | 0.0064 | 7.91E-14 | 0.6059 |
| HbA1c | rs7115739 | 11 | G | T | 0.0655 | 0.0087 | 3.85E-14 | 0.8331 |
| HbA1c | rs201509902 | 11 | T | A | -0.0388 | 0.0064 | 1.43E-09 | 0.4079 |
| HbA1c | rs10830963 | 11 | G | C | 0.065 | 0.0063 | 4.39E-25 | 0.4318 |
| HbA1c | rs1984564 | 12 | G | A | 0.1163 | 0.016 | 4.00E-13 | 0.0386 |
| HbA1c | rs7319456 | 13 | T | C | -0.0398 | 0.0064 | 5.90E-10 | 0.5468 |
| HbA1c | rs73579370 | 13 | C | T | 0.0749 | 0.0115 | 7.04E-11 | 0.089 |
| HbA1c | rs35591 | 16 | G | C | 0.0445 | 0.0063 | 1.24E-12 | 0.4686 |
| HbA1c | rs2608604 | 16 | G | A | 0.0932 | 0.0065 | 3.68E-46 | 0.6375 |
| HbA1c | rs7206953 | 17 | C | T | -0.0418 | 0.0067 | 4.18E-10 | 0.6148 |
| HbA1c | rs34299509 | 17 | GA | G | 0.0468 | 0.0066 | 1.54E-12 | 0.5109 |
| HbA1c | rs2285644 | 17 | A | G | 0.0866 | 0.0138 | 3.95E-10 | 0.0535 |
| HbA1c | rs3834968 | 17 | AG | A | 0.1589 | 0.0085 | 1.80E-78 | 0.1659 |
| HbA1c | rs113373052 | 17 | T | C | 0.0928 | 0.0063 | 9.93E-50 | 0.4749 |
| HbA1c | rs59616136 | 19 | A | G | -0.0923 | 0.0067 | 6.89E-43 | 0.3361 |
| HbA1c | rs8101428 | 19 | C | T | 0.0463 | 0.0064 | 4.49E-13 | 0.5156 |
| HbA1c | rs6099616 | 20 | C | T | 0.0451 | 0.0063 | 1.02E-12 | 0.4422 |
| HbA1c | rs855791 | 22 | G | A | -0.0611 | 0.0062 | 9.11E-23 | 0.4891 |

Chr, chromosome; EAF, effect allele frequency; FG, fasting glucose; FI, fasting insulin; NA, not available; POS, position; SE, standard error

**Table 2**. Statistically significant genetic instruments for all fasting glucose and HbA1c obtained from the Korea Biobank Array (KBA).

| **Trait** | **Novelty** | **rsID** | **CHR** | **POS** | **Effect Allele** | **Other Allele** | **EAF** | | **Effect size (β)** | **SE** | **P-value** |
| --- | --- | --- | --- | --- | --- | --- | --- | --- | --- | --- | --- |
| FG | Novel | rs11210771 | 1 | 43444560 | C | T | 0.6385 | | 0.0291 | 0.0045 | 7.74E-11 |
| FG | Known | rs1260326 | 2 | 27730940 | C | T | 0.4517 | | 0.0776 | 0.0043 | 3.49E-73 |
| FG | Novel | rs146841961 | 2 | 37575181 | ATCTCTC | A | 0.5563 | | 0.029 | 0.0043 | 1.47E-11 |
| FG | Known | rs12712928 | 2 | 45192080 | C | G | 0.3741 | | 0.0776 | 0.0045 | 1.84E-67 |
| FG | Novel | rs1912980 | 2 | 60581855 | T | C | 0.3358 | | -0.0301 | 0.0045 | 2.67E-11 |
| FG | Known | rs12053049 | 2 | 169767148 | C | T | 0.3538 | | 0.1024 | 0.0045 | 7.24E-115 |
| FG | Known | rs3813814 | 2 | 173599819 | A | G | 0.4308 | | -0.0613 | 0.0043 | 1.71E-45 |
| FG | Novel | rs832189 | 3 | 63841942 | C | T | 0.6387 | | 0.0316 | 0.0045 | 1.62E-12 |
| FG | Novel | rs1250123 | 4 | 1264864 | G | A | 0.5721 | | 0.0261 | 0.0043 | 1.65E-09 |
| FG | Novel | rs147834269 | 4 | 6303731 | A | G | 0.0317 | | -0.0825 | 0.012 | 6.47E-12 |
| FG | Known | rs9379084 | 6 | 7231843 | A | G | 0.1622 | | -0.0348 | 0.0058 | 1.63E-09 |
| FG | Known | rs10440833 | 6 | 20688121 | A | T | 0.4618 | | 0.0511 | 0.0043 | 1.31E-32 |
| FG | Known | rs742761 | 6 | 39046655 | T | C | 0.2108 | | -0.0481 | 0.0053 | 8.76E-20 |
| FG | Known | rs11309324 | 6 | 117266057 | C | CA | 0.6005 | | 0.0334 | 0.0044 | 1.65E-14 |
| FG | Known | rs35700246 | 6 | 153421680 | G | GA | 0.8945 | | 0.0473 | 0.007 | 1.43E-11 |
| FG | Known | rs10487796 | 7 | 15063430 | A | T | 0.3224 | | -0.0672 | 0.0046 | 3.57E-49 |
| FG | Known | rs1799884 | 7 | 44229068 | T | C | 0.1822 | | 0.1201 | 0.0055 | 1.91E-105 |
| FG | Known | rs11774700 | 8 | 118220270 | C | T | 0.4191 | | -0.0648 | 0.0044 | 4.72E-49 |
| FG | Known | rs58117295 | 9 | 628916 | C | T | 0.17 | | 0.0735 | 0.0058 | 9.83E-37 |
| FG | Known | rs4237150 | 9 | 4290085 | C | G | 0.4114 | | 0.0491 | 0.0044 | 2.15E-29 |
| FG | Known | rs10965248 | 9 | 22132878 | C | T | 0.4375 | | -0.0653 | 0.0043 | 7.81E-52 |
| FG | Known | rs9411372 | 9 | 136134068 | A | G | 0.256 | | 0.0335 | 0.0049 | 8.44E-12 |
| FG | Novel | rs148829035 | 10 | 26499911 | GCA | G | 0.3142 | | -0.0319 | 0.0048 | 2.91E-11 |
| FG | Novel | rs10882288 | 10 | 95389876 | T | C | 0.4543 | | -0.0262 | 0.0043 | 9.34E-10 |
| FG | Known | rs34083578 | 10 | 112989611 | T | C | 0.0424 | | -0.0737 | 0.0106 | 3.38E-12 |
| FG | Known | rs60808706 | 11 | 2857233 | A | G | 0.3925 | | -0.0576 | 0.0045 | 3.58E-37 |
| FG | Known | rs174546 | 11 | 61569830 | T | C | 0.2979 | | -0.0486 | 0.0051 | 8.39E-22 |
| FG | Known | rs148527516 | 11 | 72461812 | AG | A | 0.0613 | | -0.0604 | 0.0089 | 1.09E-11 |
| FG | Known | rs10830963 | 11 | 92708710 | G | C | 0.4318 | | 0.1021 | 0.0043 | 3.07E-125 |
| FG | Known | rs138229262 | 12 | 97853954 | C | CA | 0.438 | | -0.0297 | 0.0043 | 6.38E-12 |
| FG | Known | rs671 | 12 | 112241766 | A | G | 0.1546 | | -0.0953 | 0.0059 | 2.03E-59 |
| FG | Novel | rs4992759 | 12 | 133140409 | C | T | 0.8227 | | -0.0454 | 0.0057 | 1.85E-15 |
| FG | Known | rs34238147 | 13 | 26776255 | A | G | 0.4489 | | 0.0301 | 0.0043 | 3.24E-12 |
| FG | Known | rs3812861 | 13 | 28493489 | T | A | 0.4542 | | 0.0451 | 0.0043 | 2.60E-25 |
| FG | Known | rs2858980 | 13 | 33554587 | A | G | 0.8336 | | -0.0403 | 0.0058 | 2.52E-12 |
| FG | Known | rs7161785 | 15 | 62395224 | C | G | 0.4594 | | -0.055 | 0.0043 | 6.89E-38 |
| FG | Known | rs4965430 | 15 | 99268850 | G | C | 0.4866 | | -0.0291 | 0.0044 | 2.29E-11 |
| FG | Known | rs7219123 | 17 | 17337839 | C | G | 0.4472 | | -0.0256 | 0.0043 | 3.21E-09 |
| FG | Novel | rs2302783 | 17 | 66447073 | C | T | 0.5489 | | -0.0284 | 0.0043 | 3.04E-11 |
| FG | Known | rs1964272 | 19 | 46190268 | A | G | 0.3702 | | -0.0279 | 0.0044 | 3.71E-10 |
| FG | Known | rs1974 | 20 | 22562311 | A | G | 0.1355 | | -0.0867 | 0.0062 | 8.54E-44 |
| FG | Novel | rs1757697 | 20 | 62137971 | G | A | 0.3011 | | -0.0339 | 0.0047 | 4.59E-13 |
| HbA1c | Known | rs3093638 | 1 | 25685484 | A | G | | 0.6653 | -0.0529 | 0.0066 | 1.19E-15 |
| HbA1c | Known | rs72898974 | 1 | 50975971 | T | C | | 0.1047 | -0.0612 | 0.0102 | 1.93E-09 |
| HbA1c | Known | rs60724735 | 1 | 156297288 | C | T | | 0.2333 | -0.1019 | 0.0074 | 1.18E-43 |
| HbA1c | Known | rs2251963 | 1 | 158582543 | A | G | | 0.4154 | 0.0621 | 0.0063 | 9.02E-23 |
| HbA1c | Known | rs340515 | 2 | 45188370 | T | G | | 0.6153 | -0.0655 | 0.0065 | 4.79E-24 |
| HbA1c | Known | rs12053049 | 2 | 169767148 | C | T | | 0.3538 | 0.0712 | 0.0066 | 3.15E-27 |
| HbA1c | Known | rs1045661 | 2 | 175292888 | C | G | | 0.2349 | 0.0712 | 0.0074 | 4.42E-22 |
| HbA1c | Novel | rs507131 | 3 | 195789428 | A | C | | 0.397 | -0.0496 | 0.0064 | 8.09E-15 |
| HbA1c | Novel | rs11569142 | 4 | 110931939 | T | G | | 0.2482 | -0.0496 | 0.0073 | 1.01E-11 |
| HbA1c | Known | rs4565031 | 4 | 145044759 | A | G | | 0.5115 | 0.0376 | 0.0063 | 2.11E-09 |
| HbA1c | Known | rs10440833 | 6 | 20688121 | A | T | | 0.4618 | 0.0644 | 0.0063 | 1.44E-24 |
| HbA1c | Known | rs198851 | 6 | 26104632 | G | T | | 0.9565 | 0.0982 | 0.0152 | 1.06E-10 |
| HbA1c | Known | rs34046799 | 6 | 32625442 | A | C | | 0.1181 | 0.0704 | 0.0097 | 2.92E-13 |
| HbA1c | Novel | rs742761 | 6 | 39046655 | T | C | | 0.2108 | -0.0515 | 0.0077 | 2.53E-11 |
| HbA1c | Known | rs9376090 | 6 | 135411228 | C | T | | 0.3252 | -0.0838 | 0.0067 | 3.09E-36 |
| HbA1c | Known | rs1799884 | 7 | 44229068 | T | C | | 0.1822 | 0.1166 | 0.0081 | 1.66E-47 |
| HbA1c | Known | rs111754661 | 7 | 123426927 | C | T | | 0.1101 | -0.0592 | 0.01 | 3.38E-09 |
| HbA1c | Known | rs2233580 | 7 | 127253550 | T | C | | 0.0686 | 0.1031 | 0.0124 | 9.27E-17 |
| HbA1c | Known | rs4737009 | 8 | 41630405 | A | G | | 0.5199 | 0.0698 | 0.0062 | 4.31E-29 |
| HbA1c | Known | rs34432089 | 8 | 42400823 | A | G | | 0.3872 | -0.047 | 0.0064 | 3.13E-13 |
| HbA1c | Known | rs13266634 | 8 | 118184783 | T | C | | 0.3956 | -0.059 | 0.0064 | 2.61E-20 |
| HbA1c | Known | rs10965248 | 9 | 22132878 | C | T | | 0.4375 | -0.0594 | 0.0063 | 3.94E-21 |
| HbA1c | Known | rs28376933 | 9 | 110529852 | G | A | | 0.1046 | 0.0666 | 0.0104 | 1.37E-10 |
| HbA1c | Known | rs550057 | 9 | 136146597 | T | C | | 0.2591 | 0.0668 | 0.0071 | 5.55E-21 |
| HbA1c | Known | rs61848342 | 10 | 12303813 | C | T | | 0.4772 | 0.0442 | 0.0063 | 1.60E-12 |
| HbA1c | Known | rs2305196 | 10 | 71144324 | A | G | | 0.5152 | -0.0374 | 0.0063 | 3.07E-09 |
| HbA1c | Known | rs77329181 | 11 | 257928 | T | C | | 0.5262 | 0.0529 | 0.0069 | 2.66E-14 |
| HbA1c | Known | rs2237897 | 11 | 2858546 | T | C | | 0.3925 | -0.058 | 0.0068 | 2.04E-17 |
| HbA1c | Known | rs1002226 | 11 | 17405617 | T | C | | 0.6059 | -0.0481 | 0.0064 | 7.91E-14 |
| HbA1c | Known | rs7115739 | 11 | 61641717 | G | T | | 0.8331 | 0.0655 | 0.0087 | 3.85E-14 |
| HbA1c | Novel | rs201509902 | 11 | 78118700 | T | A | | 0.4079 | -0.0388 | 0.0064 | 1.43E-09 |
| HbA1c | Known | rs10830963 | 11 | 92708710 | G | C | | 0.4318 | 0.065 | 0.0063 | 4.39E-25 |
| HbA1c | Known | rs1984564 | 12 | 7090193 | G | A | | 0.0386 | 0.1163 | 0.016 | 4.00E-13 |
| HbA1c | Known | rs7319456 | 13 | 113525619 | T | C | | 0.5468 | -0.0398 | 0.0064 | 5.90E-10 |
| HbA1c | Known | rs73579370 | 13 | 114633330 | C | T | | 0.089 | 0.0749 | 0.0115 | 7.04E-11 |
| HbA1c | Known | rs35591 | 16 | 16141810 | G | C | | 0.4686 | 0.0445 | 0.0063 | 1.24E-12 |
| HbA1c | Known | rs2608604 | 16 | 88849421 | G | A | | 0.6375 | 0.0932 | 0.0065 | 3.68E-46 |
| HbA1c | Novel | rs7206953 | 17 | 8166378 | C | T | | 0.6148 | -0.0418 | 0.0067 | 4.18E-10 |
| HbA1c | Known | rs34299509 | 17 | 27182812 | GA | G | | 0.5109 | 0.0468 | 0.0066 | 1.54E-12 |
| HbA1c | Known | rs2285644 | 17 | 42328621 | A | G | | 0.0535 | 0.0866 | 0.0138 | 3.95E-10 |
| HbA1c | Known | rs3834968 | 17 | 76124810 | AG | A | | 0.1659 | 0.1589 | 0.0085 | 1.80E-78 |
| HbA1c | Known | rs113373052 | 17 | 80697458 | T | C | | 0.4749 | 0.0928 | 0.0063 | 9.93E-50 |
| HbA1c | Known | rs59616136 | 19 | 17252041 | A | G | | 0.3361 | -0.0923 | 0.0067 | 6.89E-43 |
| HbA1c | Known | rs8101428 | 19 | 46212952 | C | T | | 0.5156 | 0.0463 | 0.0064 | 4.49E-13 |
| HbA1c | Novel | rs6099616 | 20 | 55990656 | C | T | | 0.4422 | 0.0451 | 0.0063 | 1.02E-12 |
| HbA1c | Known | rs855791 | 22 | 37462936 | G | A | | 0.4891 | -0.0611 | 0.0062 | 9.11E-23 |

Chr, chromosome; EAF, effect allele frequency; FPG, fasting plasma glucose; NA, not available; POS, position; SE, standard error
